# ﻿Six new species of *Globba* L. (Zingiberales, Zingiberaceae) from the Eastern Himalayas and Northeast India

**DOI:** 10.3897/phytokeys.246.118751

**Published:** 2024-09-04

**Authors:** Ritu Yadav, Vinita Gowda

**Affiliations:** 1 Tropical Ecology and Evolution Lab, Department of Biological Sciences, Indian Institute of Science Education and Research Bhopal, 462066, Madhya Pradesh, India Indian Institute of Science Education and Research Bhopal Madhya India

**Keywords:** dancing girls ginger, Meghalaya, Mizoram, taxonomy, West Bengal

## Abstract

We describe six new species in the genus *Globba* L.: *Globbacorniculata***sp. nov.**, *Globbapaschimbengalensis***sp. nov.**, *Globbapolymorpha***sp. nov.**, *Globbatyrnaensis***sp. nov.**, *Globbajanakiae***sp. nov.**, *and Globbayadaviana***sp. nov.** collected from the Indian part of the Eastern Himalayas (West Bengal) and Northeast India. We provide a detailed morphological description of all six species along with photographic plates, distribution maps, and tentative conservation assessments. We also provide a dichotomous identification key for all the Indian *Globba* species and discuss the newly described species in relation to those that are morphologically similar to them. Finally, we highlight the taxonomic collection challenges in the ecologically sensitive Eastern Himalayas and Northeast regions of India.

## ﻿Introduction

**Figure 1.**
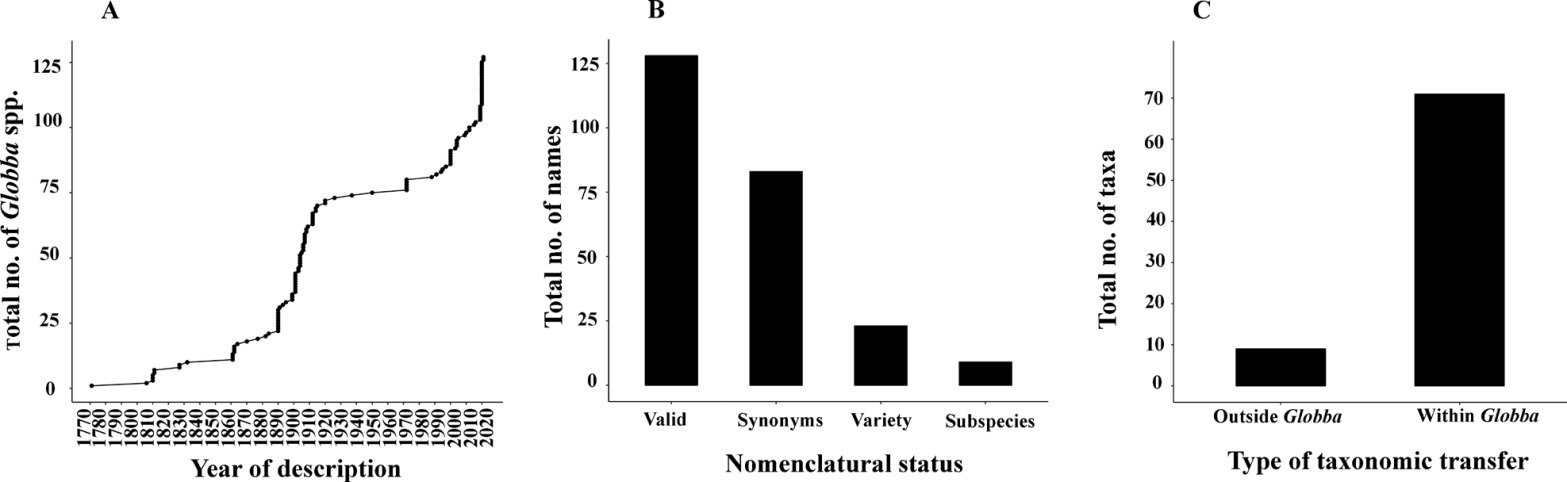
**A** yearly trend in the number of reported *Globba* spp. **B, C** nomenclatural status (**B**), and type of taxonomic transfer (**C**) in *Globba*.

Zingiberaceae is the largest family within the order Zingiberales, which comprises at least 114 genera and 4022 species (https://www.worldfloraonline.org/). Members of Zingiberaceae are tropical, perennial, rhizomatous herbs that usually grow in moist shady places (Saha et al. 2020). *Globba* L. is the fourth largest genus with about 136 species (POWO 2024; Fig. 1A) within Zingiberaceae, and it is one of the three genera within the tribe Globbeae, the other two being *Gangnepainia* K.Schum. and *Hemiorchis* Kurz (Cao et al. 2019). The genus *Globba* is distinguished from the other two genera by the presence of anther appendages, the absence of central stripe/point on labellum, the labellum being partially fused with the floral tube or free, the reflexed floral tube and flowering throughout the rainy season (Williams et al. 2004). Species within this genus are popular as ornamental plants, often known as: dancing girls, weeping goldsmith, snowball, singapore gold, white dragon, and ruby queen, all of which highlight the attractive and delicate flowers. The type species for the genus *Globba* - *G.marantina* L. was described by Linnaeus in 1771, and subsequent exploration of this genus can be accorded to Smith (1805) and Roxburgh (1820), followed by more regional-level studies throughout Southeast Asia (Sakai et al. 1999; Takano and Okada 2003; Williams et al. 2004; Sakai and Nagamasu 2006; Sakai et al. 2013; Sangvirotjanapat et al. 2019, 2020, Newman and Sangvirotjanapat 2023). Most *Globba* spp. are terrestrial, a few are lithophytes, and only one species is known to be epiphytic (*G.bokorensis* Nob.Tanaka & Tagane). Morphologically, vegetative traits do not vary as much as reproductive traits, as most of the species are small herbaceous plants with short stature and an understory growth habit. However, there is a high diversity in their reproductive traits, such as in inflorescence type and size, floral morphology (lateral staminode, labellum), presence of andromonoecy, and presence and morphology of bulbils. The flower is characterized by a long, curved filament with a terminal anther, having zero, two, four, or six appendages. Stigma is present between the anther lobes, and style is held in the ventral furrow of the filament. The anther appendages have been shown to be an important morphological trait that defines infrageneric taxonomy in *Globba*, and it has also been used along with molecular markers to identify both subgeneric and sectional delimitations (Williams et al. 2004; Sangvirotjanapat et al. 2019).

**Figure 2.**
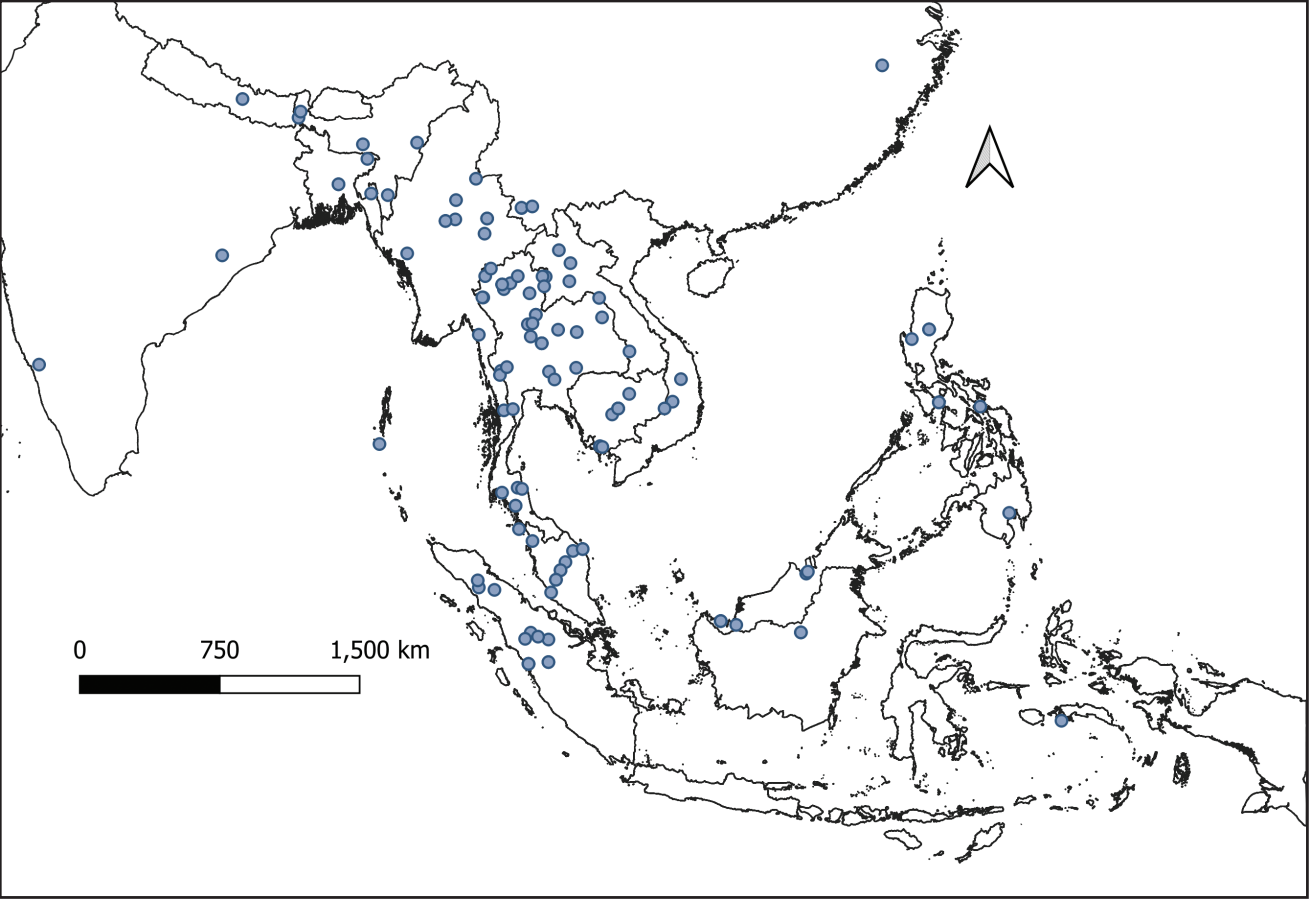
Comprehensive map showing the distribution of all type localities for *Globba* spp. in Asia.

The genus *Globba* currently includes seven sections (*Haplanthera* Horan., *Ceratanthera* (Horan.) Petersen, *Globba* (formerly G.sect.Marantella (Horan.) Benth. & Hook.f.), *Nudae* K.Larsen, *Substrigosa* K.J.Williams, *Sempervirens* K.J.Williams, and *Mantisia* (Sims) K.J.Williams). It is distributed in Sri Lanka, India, Nepal, Bhutan, Bangladesh, tropical China, and all of Southeast Asia (Fig. 2) and can be found as far east as Australia and Solomon Islands.

In recent years (2019–2022, Fig. 1A), an increased field exploration in Southeast Asia has led to a drastic increase in the number of species, making this region truly the center of diversity for this genus. In India, a total of 19 species (Suppl. material 1) have been documented, all of which are confined to the tropical forests of the Western Ghats across four states (Maharashtra, Karnataka, Kerala, and Tamil Nadu), the seven states of Northeast India (Arunachal Pradesh, Assam, Meghalaya, Nagaland, Manipur, Mizoram, and Tripura), Sikkim, and West Bengal (Alfred et al. 2019; Dalisay et al. 2022).

Based on molecular phylogenetic studies (Williams et al. 2004), eight of the Indian species have been assigned to the following three sections: *Haplanthera* (five species: *G.multiflora* Wall. ex Baker, *G.racemosa* Sm., *G.sessiliflora* Sims, *G.orixensis* Roxb., *G.macroclada* Gagnep.), *Ceratanthera* (one species: *G.pendula* Roxb.), and *Globba* (two species: *G.marantina* and *G.schomburgkii* Hook.f.). There are eight *Globba* species described from different parts of India. Williams Roxburgh described the first species from India, *G.orixensis* from Odisha, in 1810 (Roxburgh 1810), and the most recently published species, *G.kanchigandhii* A.Joe & M.Sabu is from Nagaland in 2019 (Alfred et al. 2019).

The northeast region of India is geographically seen as a ‘gateway’ for much of India’s flora and fauna because it shares its geographic borders with China and Bhutan to the north and Bangladesh and Myanmar to the south and southeast (Chakravarty et al. 2012; Ashokan et al. 2022). It has been shown for the genus *Hedychium* J.Koenig (Zingiberaceae) that the multistage uplift of the Himalayas created new geophysical environments along with climatic changes such as an intensification of monsoons which led to high speciation rates in these newly created ecological niches (Ashokan et al. 2022).

**Figure 3.**
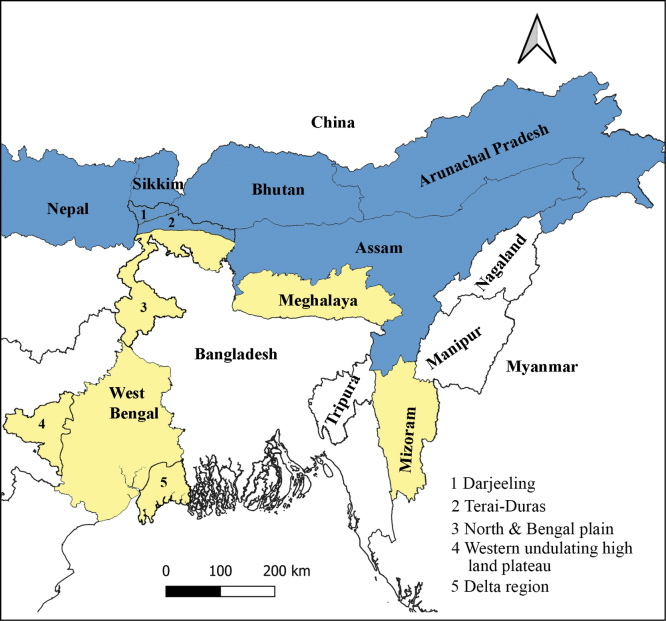
Map illustrating the Eastern Himalayas hotspot zone (highlighted in blue) and the various states of Northeast India. The states from which species are described in this study are highlighted in yellow. Additionally, the state of West Bengal is divided into five geographical regions, represented by the numbers 1, 2, 3, 4, and 5.

Similarly, other gingers in the northeast region of India may also have witnessed high speciation events, making this region a unique habitat for diverse flora with a high level of endemism (Chatterjee et al. 2006; Ashokan et al. 2022). Based on floristic composition and local climate, the northeast region of India can be divided into two biogeographic zones: Eastern Himalaya and Northeast India (Fig. 3 based on Rodgers and Panwar 1988). The Eastern Himalaya region includes (from west to east) the Indian states of West Bengal (five northern districts including Darjeeling), Sikkim, and Arunachal Pradesh and the country Bhutan, and the region of Northeast India includes the states of Assam, Meghalaya, Nagaland, Manipur, Mizoram, and Tripura (Fig. 3, Chatterjee et al. 2006).

Both of these regions have distinct climates, geology, topography, and floristic history. The state of West Bengal is further divided into five geographical regions: the Darjeeling, the Terai-Duars, the Western undulating highland plateau, the North & Bengal plain, and the Delta (Fig. 3). Among these five vegetational zones, the Darjeeling-Himalayan zone belongs to the Eastern-Himalayan Hotspots zone, and it is the richest floristic zone of the state (Mitra et al. 2021). In this study, we describe six new species of the genus *Globba* from the states of West Bengal (Darjeeling region), Meghalaya, and Mizoram in India.

## ﻿Materials and methods

Floristic surveys were conducted in the state of West Bengal, Meghalaya, and Mizoram during the monsoon months of August and September 2022, which is the flowering season for most of the *Globba* spp., and these surveys were also used to formulate the conservation assessment for all the six species described here. Morphological measurements for all six species were taken in situ from freshly dissected specimens, and from pickled flowers kept in Copenhagen solution using a digital caliper and under a stereomicroscope (Leica S8 APO), and also from digital images using the ImageJ software (Schneider et al. 2012). We also took morphological measurements from protologues, type specimens, and all available herbarium vouchers of the morphologically similar taxa (that is allied species) for comparative morphometrics with our new species. We have measured the flower length from the base of the ovary to the tip of the anther for all six newly described species. All herbarium vouchers and pickled specimens are deposited at BHPL (IISER Bhopal), and duplicates will be deposited at ASSAM (BSI Shillong). We examined specimens of *Globba*, including relevant type materials from the digitized herbarium collections of various international herbaria, such as E, K, and the JSTOR Global Plants collection (https://plants.jstor.org/), and all relevant *Globba* protologues were studied. The conservation status of the newly described species was evaluated based on guidelines listed by the International Union for Conservation of Nature (IUCN 2022), and terminologies for describing different morphological characters follow Gowda et al. 2012.

## ﻿Taxonomic treatment

### ﻿ Globba corniculata

Taxon classificationPlantaeZingiberalesZingiberaceae

Y.Ritu & V.Gowda sp. nov.

F73008B1-E9C2-526E-849D-0F353B2087B3

urn:lsid:ipni.org:names:77347881-1

Fig. 4

#### Type.

**India. West Bengal**: Darjeeling district, Takdah forest, 27.0493, 88.3555, elevation 1220 m, 20 August 2022, *Y. Ritu, S. Goray & Rhuthuparna S. B*. *VG2022WB3803* (holotype: BHPL!; isotype: ASSAM!).

#### Diagnosis.

**Figure 4.**
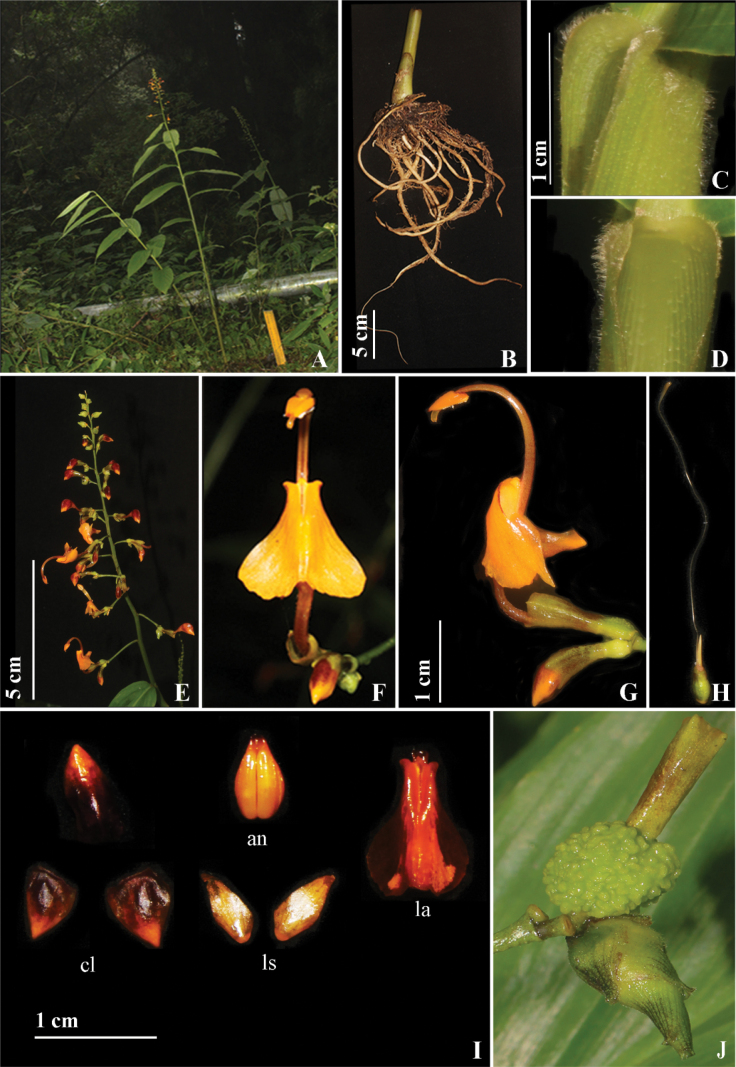
*Globbacorniculata* sp. nov. **A** habit **B** rhizome **C, D** ligule **E** inflorescence **F** flower (front view) **G** flower (side view) **H** gynoecium **I** dissected flower, cl (corolla lobes), an (anther), ls (lateral staminodes), la (labellum) **J** fruit. Photographs by Y. Ritu.

*Globbacorniculata* is morphologically similar to *G.ruiliensis* and *G.multiflora* but differs in having sessile, oblong-narrowly ovate leaves, absence of inflorescence bracts and bracteoles, orange flower, cuneate labellum with cornicula (Fig. 4F), long pedicel, the presence of bulbils throughout the inflorescence vs. petiolate, ovate to narrowly ovate leaves, presence of inflorescence bracts and bracteole, yellow to orange flowers, and obcuneate labellum with cornicula.

#### Description.

Terrestrial herbs, 100–160 cm tall including inflorescence height, pseudostem swollen at the base. Rhizomes compact, non tuberulous, creamish white. Leafy shoot with 9–12 leaves; sheath ligulate, ligule 3 mm long, bilobed, margin densely pubescent; lamina 25–32 × 6–10 cm, sessile, oblong–narrowly ovate, base rounded, apex caudate, margin entire, adaxially pubescent along veins and margins, abaxially densely pubescent. Inflorescence terminal to the leafy shoot, 25–53 cm long, erect; peduncle 18–23 mm long, light green, glabrous; rachis, dark green, glabrous, with white dots, bracts and bracteole absent. Ebracteate cincinni 25–51, flowers maturing from base to apex of inflorescence, each cincinni with 2–3 flowers. Floral pedicel for basal flowers 2.2–2.7 cm long, for terminal flowers 0.4–0.6 cm long, dark green, white dotted, glabrous. Flowers 4.2–4.9 cm long, orange, recurved; calyx 9–10 mm long, pale yellow with maroon patches; floral tube 1.5–1.8 cm long, dark orangish red, densely pubescent, curved upwards holding the flower upright; dorsal corolla lobe 8–9 × 3–4 mm, ovate, apex cucullate, dark maroon with orange tip, densely pubescent; lateral corolla lobes 7–8 × 5–5.5 mm, ovate, apex acute, dark maroon with orange apex, glabrous; labellum 1.3 × 0.6–0.8 cm, cordate, corniculate, orange, linear structures scattered on ventral surface; lateral staminodes 9–10 × 3–4 mm, narrowly ovate, apex attenuate, orange, glabrous. Stamen 2.1–2.4 cm long, filament 1.5–1.8 cm long, orange tinged red, glabrous, fulvous with red dots, arching; anther 5–6 mm long, ovate, apex acute, orange tinged red, crest 0.8–0.9 mm long, non-appendaged; style 4.3–4.8 cm, filiform, apex broader than base, stigma 4–5 mm long, cylindrical, apex ciliate. Ovary 0.4–0.5 × 0.3 cm, obovate, pale green, verrucose; epigynous glands 2, 0.4–0.5 cm long, linear, cream. Fruit 1.5 cm in diameter, globose, green, verrucose, calyx persistent. Bulbils 1.2–2 × 0.5–0.8 cm, narrowly ovoid, yellowish green with dark maroon lines, pubescent, bulbils often at the base within a cincinni, but present throughout the inflorescence.

#### Distribution and ecology.

At present, *G.corniculata* is known only from the Takdah Forest in West Bengal, which is a tropical evergreen, lower montane forest at an elevation of ca. 1220 m. It is terrestrial, mostly growing in open habitats along the edge of roads. The current location has ca. 70 individuals within an area of ca. 400 m^2^. Other sympatric ginger species were *G.racemosa* and *Hedychiumspicatum* Sm. We have observed several species of bees and butterflies visiting the flowers of this species.

#### Phenology.

Flowering and fruiting were observed in the month of August.

#### Etymology.

The species epithet ‘corniculata’ refers to the presence of horn-shaped structure at the base of labellum.

#### Informal conservation status.

*G.corniculata* is known only from one population, which is a protected area with limited or no anthropogenic disturbance. The population was spread across 2–3 km along the edge of the road, where we counted 1500–2000 individuals. We surveyed six potential locations in Darjeeling, which is 50 km^2^ around the type locality (Takdah forest) and could not locate any population in northern West Bengal. Based on the IUCN (2022) guidelines, we hypothesize that if a formal conservation assessment were performed, its conservation status would probably be Critically Endangered according to criteria B1. (a) (B1: extent of occurrence is less than 100 km^2^, a: = 1 location).

#### Specimens of allied species examined.

***G.ruiliensis*: China. Yunnan Province**: Ruili City, Nongdao Town, Dengga village, 23.95 N, 97.55 E, elevation 854 m, 21 October 2019, *Jian-Yong Shen, Wen-Guang Wang & Xing-Da Ma 1666* (holotype: HITBC!)

***G.multiflora*: India. Sikkim**: Regio Trop, (PL0092 1837), (P01743164). **Assam**: Khasia Hills (L0487990), Khasi Hills, Nambar Forest, 3 July 1949, elevation 91.44 m, *F. Kingdon-Ward 18619* (NY02650821), *Jenkins F Jenkins, F s.n.* (L0487989).

#### Taxonomic notes.

The new species is similar to the two species in flower color and presence of cornicula at the base of the labellum. The new species differs from *G.ruiliensis* in its large inflorescence 25–53 cm (vs. 10–35 cm), short peduncle 1.8–2.3 cm (vs. 3–15 cm), glabrous rachis (vs. hirsute) and absence of inflorescence bracts (vs. present). The new species differs from *G.multiflora* in its large lateral staminodes 9–10 mm (vs. 3–4 mm), length of lateral staminodes equal to corolla lobes (vs. shorter than corolla lobes), and production of bulbils throughout the inflorescence (vs. only at the lower portion of inflorescence). The detailed morphological comparisons between *G.corniculata* and *G.ruiliensis*, *G.multiflora* are presented in Table 1.

**Table 1.** Comparison of morphological characteristics of *G.corniculata* sp. nov. with *G.ruiliensis* and *G.multiflora*. Characters not described in the original protologue or in subsequent descriptions of the same species are alternatively marked as not known.

Characters	*G.corniculata* Y.Ritu & V.Gowda	*G.ruiliensis* X.D.Ma, W.G.Wang & J.Y.Shen (Ma et al. 2021)	*G.multiflora* Wall. (Alfred et al. 2019, Lal and Verma 1987)
Ligule length (mm) and indumentum	3, densely pubescent	2, margin ciliate	1–1.6, pubescent
Lamina	Sessile	Subsessile or shortly petiolate	Sessile
Peduncle length (cm) and indumentum	1.8–2.3, glabrous	3–15, hirsute	4.9–5.6, densely pubescent
Inflorescence length (cm) and orientation	25–53, erect	10–35, erect	9.5–25, erect
Rachis indumentum	Glabrous	Hirsute	Not known
Inflorescence bracts	Absent	Present	Absent
Calyx length (mm)	9–10	7–9	8–9
Flower color	Orange	Yellow to orange	Orange
Labellum dimensions (mm) and shape	13 × 6–8, cordate	Not known, obcuneate	8–10, obcuneate
Labellum color	Orange	Yellow to orange	Saffron yellow with two red blotches
Lateral staminode dimensions (mm) and shape	9–10 × 3–4, ovate, apex attenuate	7–8 × 3–4, ovate-oblong, apex rounded	3–4 × 1.5–2, ovate-narrowly ovate, apex acute
Length of lateral staminodes with respect to corolla lobes	Nearly equal to corolla lobes	Nearly equal to corolla lobes	Shorter than (half the length) to corolla lobes
Filament length (cm)	1.5–1.8	1.2–1.7	1.5–1.7
Anther length (mm) and shape	5–6, ovate, apex acute	3–4, elliptic, apex acute	4, narrowly ovate, apex capitate
Anther crest length (mm)	0.8–0.9	Not known	Minutely crested
Ovary dimensions (mm) and shape	3.7–4.5 × 2.6–3, obovoid	4–5 × 2.5, oblong	3
Bulbil position	Throughout the inflorescence	2–4 at the lower part of inflorescence	3–5 at the lower part of inflorescences

#### Notes.

**Figure 5.**
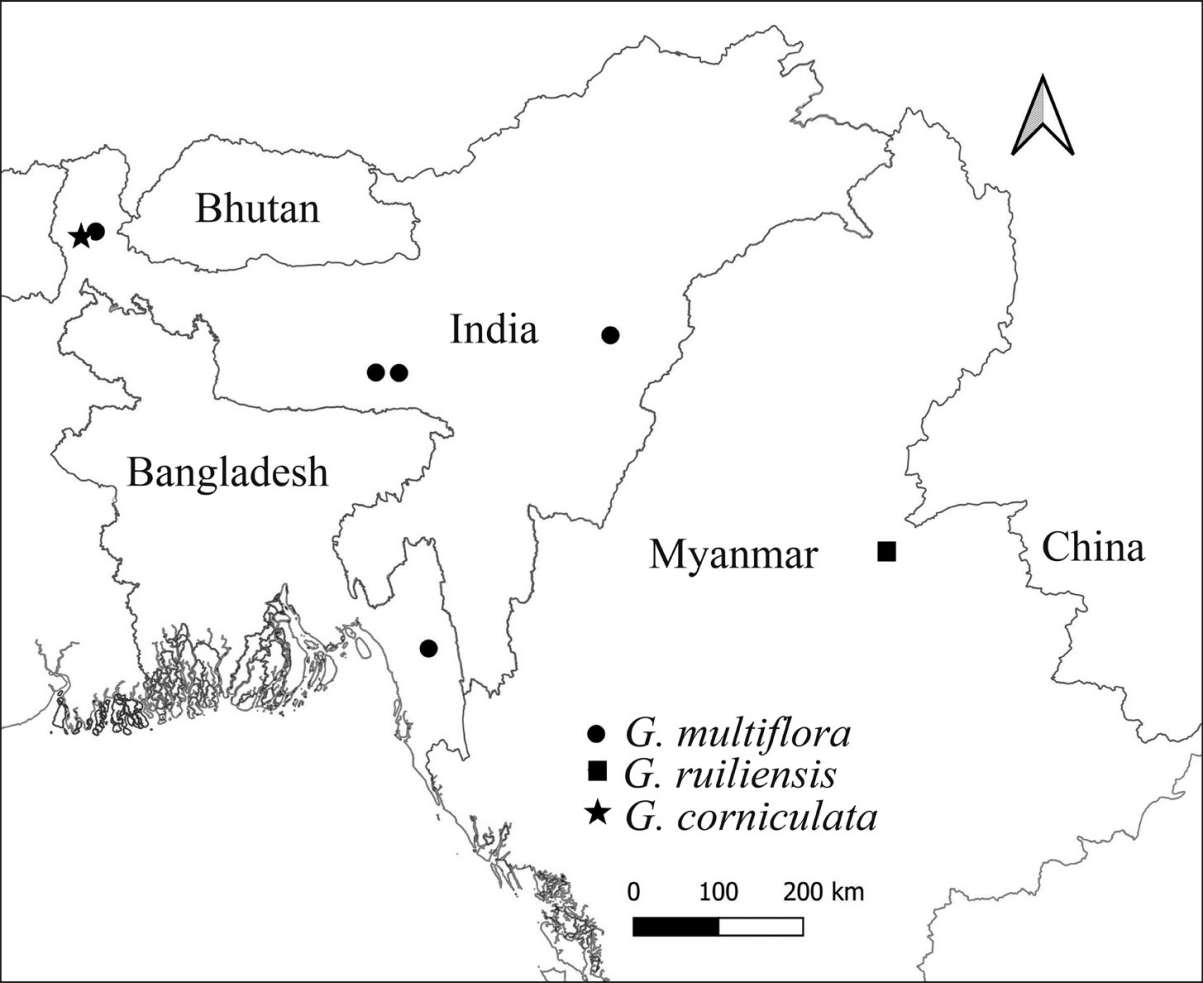
Map showing the distribution of *G.multiflora*, *G.ruiliensis*, *and G.corniculata* sp. nov.

*G.ruiliensis* is distributed in Yunnan province, China and *G.multiflora* is distributed in Meghalaya, Assam, and Sikkim states, India and Bangladesh (Fig. 5).

### ﻿ Globba paschimbengalensis

Taxon classificationPlantaeZingiberalesZingiberaceae

Y.Ritu & V.Gowda sp. nov.

D54DC9F6-2D0A-5894-B3AE-8A199C2291B4

urn:lsid:ipni.org:names:77347882-1

Fig. 6

#### Type.

**India. West Bengal**: Darjeeling district, Latpuncher, 26.9159, 88.4028, elevation 1200 m, 26 August 2022, *Y. Ritu, S. Goray VG2022WB3852* (holotype: BHPL!; isotype: ASSAM!).

#### Diagnosis.

**Figure 6.**
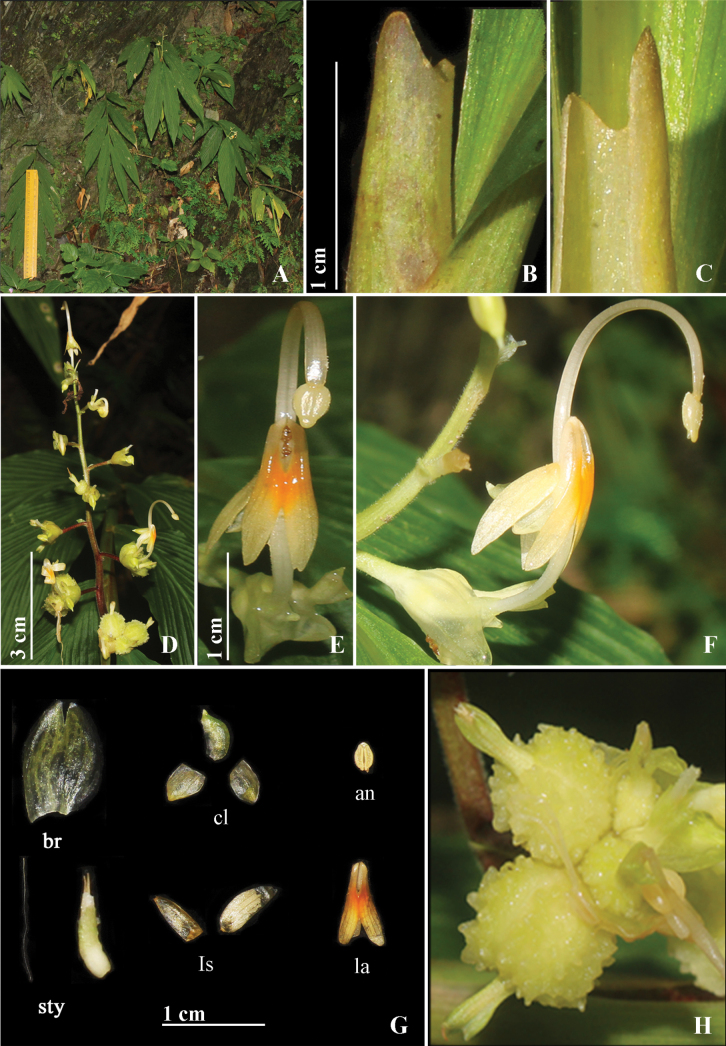
*Globbapaschimbengalensis* sp. nov. **A** habit **B, C** ligule **D** inflorescence **E** flower (front view) **F** flower (side view) **G** dissected flower, br (bracteole), cl (corolla lobes), an (anther), sty (style with stigma, ovary and epigynous gland), ls (lateral staminodes), la (labellum) **H** fruit. Photographs by Y. Ritu.

*G.paschimbengalensis* is morphologically similar to *G.andersonii* but differs in having off-white flowers with a faint tinge of yellow, deeply notched ligule with unequal lobes (Fig. 6C) reduced or no peduncle vs. white flowers, ligule with equal lobes and with peduncle.

#### Description.

Lithophyte herbs, 50–70 cm tall including inflorescence height, pseudostem swollen at the base. Rhizomes compact, non-tuberulous, creamish-white. Leafy shoot with 10–12 leaves; sheath ligulate, ligule 0.9–1.1 cm long, bilobed, lobes unequal, margin minutely pubescent; lamina 24–30 × 4–8 cm, sessile, narrowly ovate, base obtuse, apex caudate, margin entire, adaxially pubescent along veins and margins, abaxially glabrous, veins prominent. Inflorescence terminal to the leafy shoot, 8–12 cm long, erect; peduncle absent or reduced; rachis, dark maroon at the base and terminally light green, densely pubescent; bracts 1.6–1.7 × 1.4–1.5 mm, obovoid, pale yellow, pubescent adaxially, glabrous abaxially, persistent; bracteole 5.5–8.3 × 1.9–7.3 mm, ovoid, pale yellow, glabrous. Cincinni 7–10 per inflorescence, each cincinni with 2–5 flowers. Floral pedicel for basal flowers 2–2.5 cm long, for terminal flowers 0.2–0.4 cm, dark maroon, densely pubescent. Flowers 4.2–4.5 cm long, pale dull yellow, recurved; calyx 5–6 mm long, gray-green; floral tube 8.3–10 mm long, off-white tinged yellow, densely pubescent, curved upwards holding the flower upright; dorsal corolla lobe 5–6 × 2.4–2.5 mm, ovate, apex cucullate, off-white tinged green, sparsely pubescent; lateral corolla lobes 4.5–5 × 3–4 mm, ovate, apex acute, off-white tinged green, glabrous; labellum 9–10 × 2.4–2.5 mm, decurrent, sagittate, off-white with orange spot in center, glabrous, labellum notch with echinate structures; lateral staminodes 7.8–8.8 × 2.6–3.2 mm, narrowly ovate, apex acute, off-white tinged faintly yellow, glabrous. Stamen 2.4–2.5 cm long, filament 1.9–2 cm long, off-white tinged yellow, glabrous, arching; anther 4–5 mm long, largely ovate, off-white, crest 0.4 mm long, obtuse, non-appendaged; style filiform, apex broader than base, stigma 3–4 mm long, cylindrical, apex ciliate. Ovary 1.7 × 1.3 mm, ovoid, cream; epigynous glands 2, 1.2–1.2 mm, linear, cream. Fruit 1.5 cm in diameter, globose, greenish yellow, verrucose, calyx persistent. Bulbils absent.

#### Distribution and ecology.

*G.paschimbengalensis* is recorded only from Latpuncher, Darjeeling district, West Bengal, where we observed ca. 30 individuals in an area of an estimated 400 m^2^. *G.paschimbengalensis* is lithophytic, mostly growing along the edge of roads at an elevation of ca. 1200 m. We have observed bumblebees visiting the flowers of this species.

#### Phenology.

Flowering and fruiting were observed in the month of August.

#### Etymology.

The species epithet refers to the Indian state of West Bengal, where this species was found.

#### Informal conservation status.

We have only found one population that was not in a protected area. The population was spread across a 20–25 m^2^ area with a total of 60–70 individuals only. We surveyed six potential locations in Darjeeling in a 40 km^2^ area around the type locality (Latpuncher) and did not find any population in northern West Bengal. Based on the IUCN (2022) guidelines, we informally assess the status as Critically Endangered according to criteria B1. (a) (B1: extent of occurrence is less than 100 km^2^, a: =1 location), and C. (C: fewer than 250 mature individuals).

#### Specimens of allied species examined.

***G.andersonii*: India. West Bengal**: Darjeeling Himalaya, around Baghpul, elevation 200–300 m, 6 July 2011, *S. Nirola & AP Das 1334A* (holotype: CAL!), Sivok Hill Forest, Near Coronation Bridge in the ghat region, 2 July 2011, *Sachin A. Punekar s.n.* (CALI!), elevation 914 m, 15 July 1913 (E00095574), Mongpoo, elevation 914.4 m, 12 July 1884, *Williams* (P00411420), Mongpoo, 914.4 m, 12 July 1884, *Williams* (P00252245), Darjeeling, Pankabari, elevation 762 m, July 1874, *J. S. Gamble 8130* (K000640559). **Sikkim**: elevation 305 m, 6 July 1915 (E00095573), Regio Trop (P00686468).

#### Taxonomic notes.

The new species is similar to one species in inflorescence length, presence of inflorescence bracts, and absence of bulbils. This species differs from *G.andersonii* in having unequal lobes, deeply notched ligule (vs. equal lobes and slightly notched), absence of peduncle (vs. presence of peduncle), and large lateral staminodes 7.8–8.8 mm (vs. 5–6 mm). The detailed morphological comparisons between *G.paschimbengalensis and G.andersonii* are presented in Table 2.

**Table 2.** Comparison of morphological characteristics of *G.paschimbengalensis* sp. nov. and *G.polymorpha* sp. nov. with *G.andersonii*. Characters not described in the original protologue or in subsequent descriptions of the same species are alternatively marked as not known.

Characters	*G.paschimbengalensis* Y.Ritu & V.Gowda	*G.polymorpha* Y.Ritu & V.Gowda	*G.andersonii* C.B.Clarke ex Baker (according to Nirola and Das 2015, Thachat et al. 2020)
Ligule length (mm) and indumentum	0.9–1.1, minutely pubescent, unequal lobes, deeply notched	1.5–1.8, densely pubescent, deeply notched at center	0.8–1.2, pubescent externally, entire, slightly notched at center
Lamina	Sessile	Sessile	Sessile
Peduncle length (cm) and indumentum	Absent	1.56, densely pubescent	Densely pubescent
Inflorescence length (cm)	8–12	7.5–16	8–16
Bract dimensions (mm), shape, and color	1.6–1.7 × 1.4–1.5, obovate, pale green	4.6–9.2 × 2.2–3.4, elliptic, olive green with brown tinge	5–6 × 1.5–2, narrowly ovate, pale green
Calyx length (mm) and color	5–6, gray green	4–5, maroon	5–6, greenish
Flower color	Off-white with a hint of yellow	Pale dull yellow	White
Lateral staminode dimensions (mm) and shape	7.8–8.8 × 2.6–3.2, narrowly ovate	6–6.5 × 2–2.5, narrowly ovate	5–6 × 2, narrowly ovate
Length of lateral staminodes with respect to corolla lobes	Longer than corolla lobes	Slightly longer than corolla lobes	Slightly longer than corolla lobes
Filament length (cm) and color	1.9–2, off-white with a hint of yellow	1.6–1.8, off white with a hint of yellow	1.5–1.8, white
Anther length (mm) and color	4–5, gray green	2.5–3, pale yellow	1.5 × 1, white
Anther crest length (mm)	0.4	0.3	Not known
Ovary dimensions (mm)	1.7 × 1.3	2.9 × 1.7	3 × 2
Bulbil	Absent	Absent	Absent

### ﻿ Globba polymorpha

Taxon classificationPlantaeZingiberalesZingiberaceae

Y.Ritu & V.Gowda sp. nov.

7147D5BF-591B-5E82-8378-213C2788C49F

urn:lsid:ipni.org:names:77347883-1

Fig. 7

#### Type.

**India. West Bengal**: Darjeeling district, Pankhabari, 26.8326, 88.2662, elevation 600 m, 6 September 2022, *Y. Ritu, & P. A. Shangreiphao VG2022WB3906* (holotype: BHPL!; isotype: ASSAM!).

#### Diagnosis.

**Figure 7.**
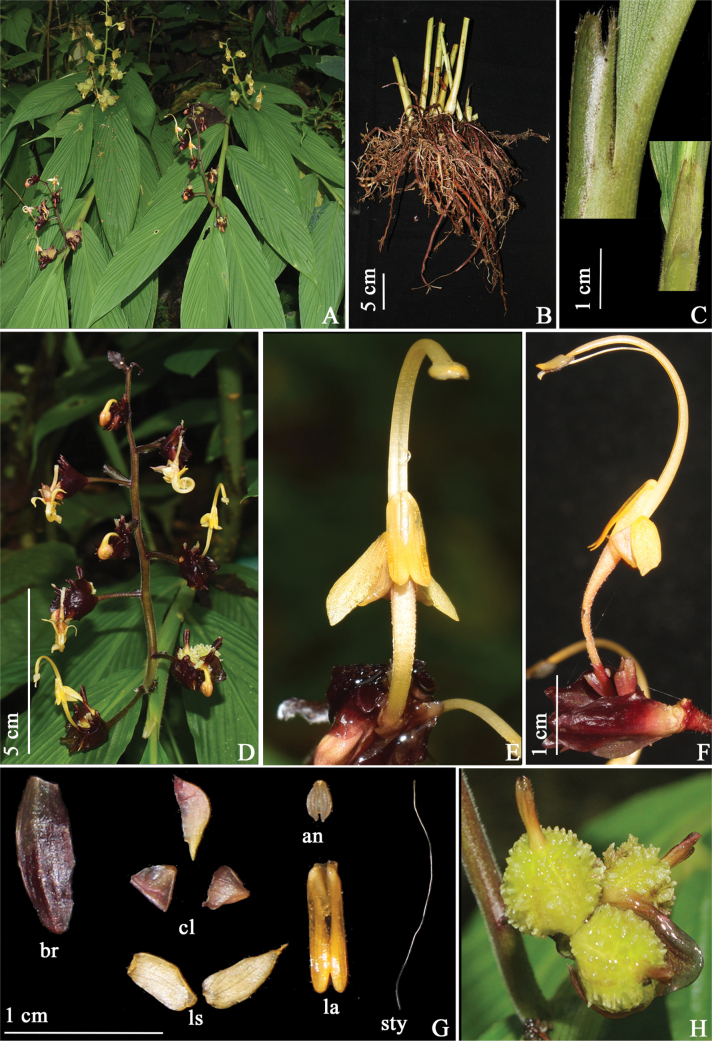
*Globbapolymorpha* sp. nov. **A** habit **B** rhizome **C** ligule **D** inflorescence **E** flower (front view) **F** flower (side view) **G** dissected flower, br (bracteole), cl (corolla lobes), an (anther), ls (lateral staminodes), la (labellum), sty (style and stigma) **H** fruit. Photographs **A, D** and **E** by Rhuthuparna SB, rest by Y. Ritu.

*G.polymorpha* is morphologically similar to *G.andersonii* but differs in having pale yellow flower, densely pubescent ligule with long white hairs (Fig. 7C), red or green-colored bracteole, large anther (4–5 mm) vs. white flowers, ligule pubescent externally, white color bracteole and small anther (1.5 mm).

#### Description.

Lithophytic herbs, 42–68 cm tall including inflorescence height, pseudostem swollen at the base. Rhizomes compact, non-tuberulous, creamish white. Leafy shoot with 6–12 leaves; sheath ligulate, ligule 1.5–1.8 cm long, bilobed, densely pubescent; lamina 22–28 × 5–9 cm, sessile, narrowly ovate, base attenuate, apex caudate, margin entire, adaxially pubescent along veins and margins, abaxially pubescent. Inflorescence terminal to the leafy shoot, 7.5–16 cm long, erect; peduncle 1.5 cm long, green with red dots to dark maroon, densely pubescent; rachis, green with red to dark maroon spots, densely pubescent; bracts 4.7–9.2 × 2.2–3.4 mm, elliptic, olive green with brown tinge, glabrous; bracteole 6.4–8.7 × 2.9–4.7 mm, obovoid to ovoid, brown with purple tinge, glabrous. Cincinni 7–17, each cincinni with 4–6 flowers. Floral pedicel for basal flowers 1.2–1.4 cm long, for terminal flowers 0.6–0.8 cm long, dark maroon, sparsely pubescent. Flowers 4–4.5 cm long, pale dull yellow, recurved; calyx 4–5 mm long, maroon; floral tube 8–9.3 mm long, pale yellow tinged pink, densely pubescent, curved upwards holding the flower upright; dorsal corolla lobe 5–6 × 2.5 mm, ovate, apex cucullate, light maroon, pubescent; lateral corolla lobes 3–3.5 mm × 2.2 mm, ovate, apex acute, yellow tinged maroon, pubescent; labellum 7–8 × 1.5–1.8 mm, decurrent, linear, pale dull yellow, glabrous, labellum notch with echinate structures; lateral staminodes 6–6.5 × 2–2.5 mm, narrowly ovate, apex acute, pale dull yellow, glabrous. Stamen 1.9–2.1 cm long, filament 1.6–1.8 cm long, off-white tinged yellow, glabrous, arching; anther 2.5–3 mm long, largely ovate, apex attenuate, pale yellow, crest 0.3 mm long, non-appendaged; style, filiform, stigma 4–5 mm wide, funnel-shaped, apex ciliate. Ovary 2.9 × 1.7 mm, ovoid, maroon, verrucose; epigynous glands 2, 1.2–1.3 mm, linear, cream. Fruit 1.5 cm in diameter, globose, greenish yellow, verrucose, calyx persistent. Bulbils absent.

#### Distribution and ecology.

*G.polymorpha* is recorded only from Pankhabari, Darjeeling district, West Bengal. At Pankhabari, we have observed ca. 21 individuals in a ca. 400 m^2^ area. *G.polymorpha* is lithophytic, mostly growing along the edge of roads at an elevation of ca. 600 m. We observed floral color polymorphism in this species, with red bracteole and yellow bracteole individuals growing within the same population. We have observed different species of bees and butterflies visiting the flowers of this species.

#### Phenology.

Flowering and fruiting were observed in the month of September.

#### Etymology.

The species epithet refers to the floral color variation due to bracteole color variations among individuals within the same population, which can be seen in Fig. 7A (top left and bottom left).

#### Informal conservation status.

*G.polymorpha* is known from only one population, which was not a protected habitat or area. The population was spread across a 10–15 m long stretch along the roadside with 20–25 individuals. We surveyed six potential locations in the Darjeeling district, which is 40 km^2^ around the type locality (Pankhabari), and we did not locate any population in northern West Bengal. Based on the IUCN (2022) guidelines and a formal conservation assessment based on the known distribution and number of individuals present, we propose its conservation status to be critically endangered according to criteria B1. (a) (B1: extent of occurrence is less than 100 km^2^, a: =1 location), and D. (D: number of mature individuals <50). In 2022, we observed 20–25 individuals at the type location, most of which were cleared for road extension, which led to a decrease in the number (10) of individuals, suggesting that the species is at risk of extinction.

#### Specimens of allied species examined.

***G.andersonii*: India. West Bengal**: Darjeeling Himalaya, around Baghpul, elevation 200–300 m, 6 July 2011, *S. Nirola & AP Das 1334A* (holotype: CAL!), Sivok Hill Forest, Near Coronation Bridge in the ghat region, 2 July 2011, *Sachin A. Punekar* s.n. (CALI!), elevation 914 m, 15 July 1913 (E00095574), Mongpoo, elevation 914.4 m, 12 July 1884, *Williams* (P00411420), Mongpoo, elevation 914.4 m, 12 July 1884, *Williams* (P00252245), Darjeeling, Pankabari, elevation 762 m, July 1874, *J. S. Gamble 8130* (K000640559). **Sikkim**: elevation 305 m, 6 July 1915 (E00095573), Regio Trop (P00686468).

#### Taxonomic notes.

The new species is similar to one species in inflorescence height, sessile leaves, densely pubescent peduncle, and absence of bulbils. This species differs from *G.andersonii* in its large ligule 1.5–1.8 mm (vs. 0.8–1.2 mm), pale dull yellow flowers (vs. white), and large anther 2.5–3 mm (vs. 1.5 mm). Detailed morphological comparisons between *G.polymorpha* and *G.andersonii* are presented in Table 2.

#### Notes.

**Figure 8.**
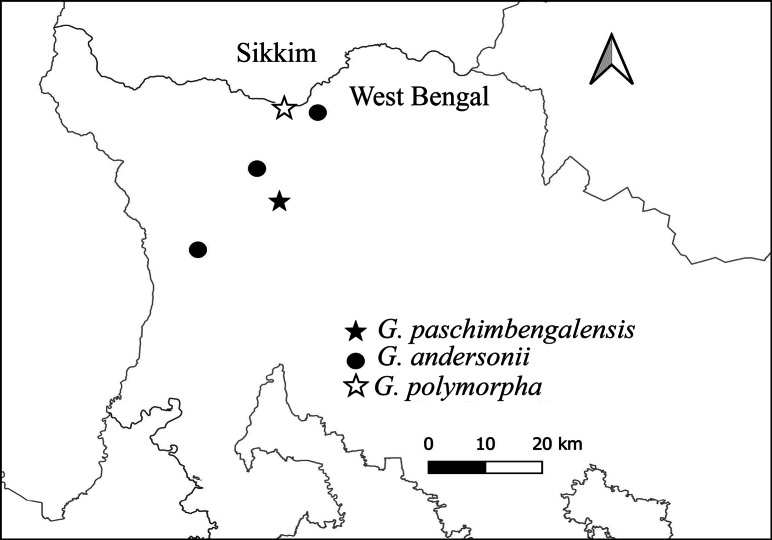
Map showing the distribution of *G.paschimbengalensis*, *G.andersonii*, *and G.polymorpha* sp. nov.

*G.andersonii* is distributed to West Bengal, India (Fig. 8).

### ﻿ Globba tyrnaensis

Taxon classificationPlantaeZingiberalesZingiberaceae

Y.Ritu & V.Gowda sp. nov.

4D48A861-15B4-521F-9640-4FEB59B7D46B

urn:lsid:ipni.org:names:77347884-1

Fig. 9

#### Type.

**India. Meghalaya**: East Khasi Hills district, Tyrna village, Double decker bridge, 25.2513, 91.672, elevation 731 m, 25 July 2022, *Y. Ritu VG2022WB3725* (holotype: BHPL!; isotype: ASSAM!).

#### Diagnosis.

**Figure 9.**
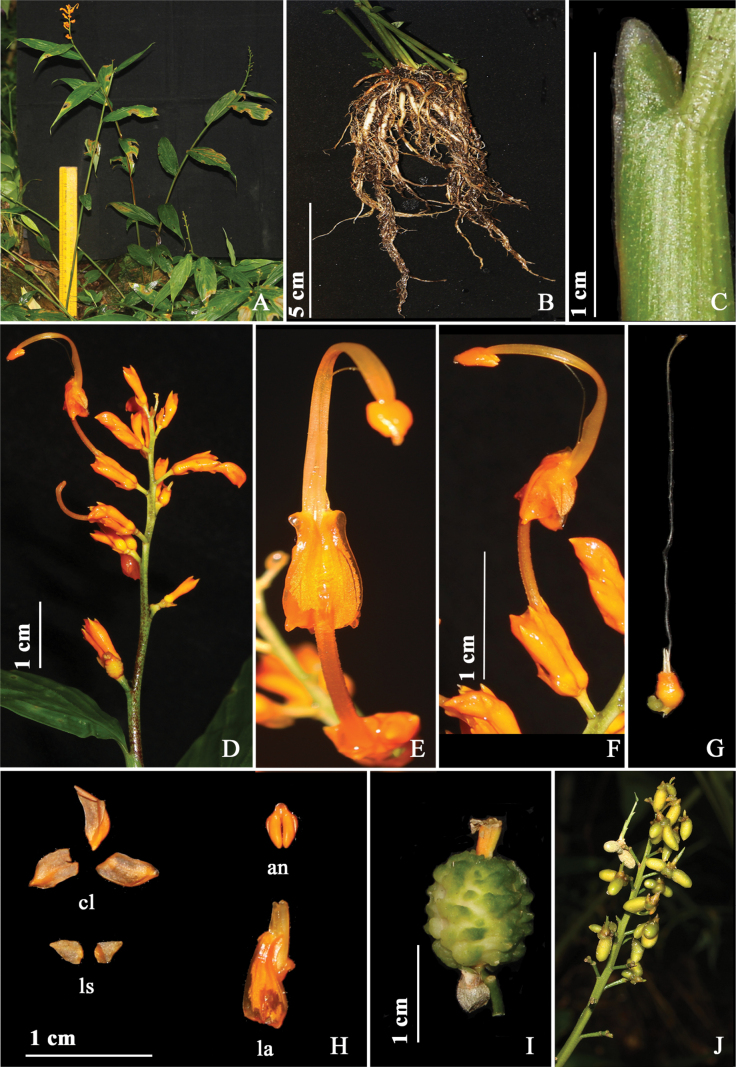
*Globbatyrnaensis* sp. nov. **A** habit **B** rhizome **C** ligule **D** inflorescence **E** flower (front view) **F** flower (side view) **G** gynoecium **H** dissected flower, cl (corolla lobes), an (anther), ls (lateral staminodes), la (labellum) **I** fruit **J** Bulbils. Photographs by Y. Ritu.

*G.tyrnaensis* is morphologically similar to *G.orixensis and G.macroclada* but different in having short inflorescence, absence of inflorescence bracts, petiolate lamina, short filament, large anther (Fig. 9) vs. large inflorescence, presence of inflorescence bracts, sessile lamina, large filament and small anther.

#### Description.

Terrestrial herbs, 32–59 cm tall including inflorescence height, pseudostem swollen at the base. Rhizomes compact, non-tuberulous, creamish-white. Leafy shoot with 6–11 leaves; sheath ligulate, ligule 3 mm long, bilobed, translucent margin and green in center, turns papery when dry, pubescent; lamina 13–18 × 2.8–3.2 cm, subsessile, petiole 0.5 cm, green with cream color stripes, glabrous, narrowly ovate, base obtuse, apex caudate, margin entire, glabrous. Inflorescence terminal to the leafy shoot, 3–8 cm long, erect; peduncle 18 mm long, light green with maroon dots, glabrous; rachis, green with maroon patches, glabrous, bracts and bracteoles absent. Ebracteate cincinni 5–16, each cincinni with 2–3 flowers. Floral pedicel 0.15–0.5 cm, light green, glabrous. Flowers 3–3.9 cm long, orange, recurved; calyx 8–9 mm long, yellowish orange; floral tube 1.2–1.3 mm long, dull orangish red, densely pubescent, bent upwards holding the flower upright; dorsal corolla lobe 5.2–5.5 × 2.3–3.2 mm, ovate, apex cucullate, orange, glabrous; lateral corolla lobes 5.7 × 3.3 mm, ovate, apex acute, yellow, glabrous; labellum 7 × 5 mm, corniculate, ovate, orange in center with lighter orange margin, labellum notch with echinate structures, cornicula 0.2–0.3 mm in length, glabrous; lateral staminodes 4.7–4.8 × 2.7 mm, narrowly ovate, apex acute, orange, hairs on margin. Stamen 2.1 cm long; filament 1.8 cm long, orange, glabrous, arching; anther 3.7 mm long, ovate, apex truncate, orange, crest 0.7–0.8 mm long, non-appendaged; style filiform, apex broader than the base, stigma 4–5 mm, cylindrical, apex ciliate. Ovary 3.1–3.6 × 2.5 mm, obovate, yellow, verrucose, epigynous glands 2, 0.3 cm long, linear, cream. Fruit 1.2 cm in diameter, globose, green, verrucose, calyx persistent. Bulbils 1.2–2 × 0.5–0.8 cm, ovoid, light green to light yellow, pubescent, bulbils present in the inflorescence and axil of leaves.

#### Distribution and ecology.

At present, we have seen *G.tyrnaensis* in the Double decker bridge, Tyrna village, and Thangkharang Park, Cherrapunji, Meghalaya. We have observed 150–200 individuals in Tyrna village and 10–15 individuals in Thangkharang Park, and the overall area of these two populations is ca. 400 m^2^ each. *G.tyrnaensis* is terrestrial mostly growing in understory habitats inside forests at an elevation of ca. 731 m. We have observed different species of bees visiting the flowers of this species.

#### Phenology.

Flowering and fruiting were observed in the month of July.

#### Etymology.

The species epithet refers to the type locality for this species, which is at the Tyrna village, Cherrapunji, Meghalaya.

#### Informal conservation status.

We have documented *G.tyrnaensis* from two populations within the state of Meghalaya: at Double decker bridge (Cherrapunji district) and near Thangkharang Park (Cherrapunji district). Both populations were in community-protected areas with limited anthropogenic disturbance. The first population was spread across 20–30 km along the edge of the road, where 300–400 individuals were counted. The second population was spread across 5–6 km with 10–15 individuals. We surveyed seven potential locations in Meghalaya, which accounts to ~50 km^2^ around the type locality (Double decker bridge), and we did not locate any other population of this species in this region. Based on the IUCN (2022) guidelines and observed population sizes and area of occupancy, we propose the conservation status of *G.tyrnaensis* to be Endangered according to criteria B1. (a) (B1: extent of occurrence is less than 100 km^2^, a: less than or equal to 5 locations).

#### Specimens of allied species examined.

***G.orixensis*: India. Assam**: Barak valley, Cachar district, Kumbhirgram, 21 July 2009, Coll.: *D. Bhattacharyya 2501*, Fl. & Fr.; Dargakona, Assam University Silchar Campus, behind Life Science and Bioinformatics Department, along the trek path to ecoforest, 24.6870 N & 92.7521E, 13 July 2010, Coll.: Fl. (Herbarium of Department of Life Science & Bioinformatics, Assam University, Silchar), *L. Darlong & D. Bhattacharyya 10063*,

28 May 1808 (E00095817), 13 June 1920 (E00095807), (E00097427), elevation 610 m, 31 May 1882 (E00097430), 13 June 1920 (E00095807), elevation 610 m, 31 May 1882 (E00097430), 28 May 1808 (E00095817), 28 May 1809 (E00095821), (E00095820), (E00097427), (E00095812), 25.583 N, 91.633 E (L0487999), *Wallich N* (L0487998), Nayagarh, 5 August 1936, *H. F. Mooney 528* (L0487991). **Odisha**: Keonjhar, *H. F. Moooney 152* (L0488000). **Meghalaya**: Khasia, Regio Trop, *Hooker J. D.* (L0488002), Regio Trop, 28 September 2001, *Hooker J. D*. (P01743148) Hort Bot. Calcutta et Serampore, *Voigt, J.O. 101* (P00252266), East Himalaya, *5634* (P01743147). **Myanmar.** 13 August 1908 (E00097420), elevation 137 m, 13 August 1909 (E00097421), 13 August 1908 (E00097420), Sagaing Division, *W. J. Kress 2-7123* (US00605376). **Bangladesh.** Elevation 40 m, 3 September 1999 (E00189266), 22.695 N, 92.237 E, elevation 40 m, 5 September 1999 (E00189267), Sreemangal, Lawachera forest, 24.25 N, 91.583 E, 8 May 1997, *Williams K J* (L0413463)

***G.macroclada*: India.***Wallichn 6411* (L0487998), **Assam**: Khasia, 25.583 N, 91.633 E, 5 August 1936, *Tea Deputation Tea Deputation s. n.* (L0487999), Nayagarh, 8 May 1997, *Mooney H. F. 528* (L0487991). **Sikkim**: East Himalaya, *Griffith W 5639* (L0041113), *Herb. Watt 8702* (E00095812), (E00097427), *6536G* (E00095820), 28 May 1808, 7 (E00095821), elevation 610 m, 31 May 1882, *6931* (E00097430), 13 June 1920, 277 (E00095807), Khasia, *Hooker J. D.* (P01743148), East Himalaya, *Herb. Griffith 5634* (P01743147), Hort. Bot. Calcutta et Serampore, *Voigt, J. O. 101* (P00252266), East Himalaya, 12 July 1884, *Griffith, W. 5639* (P032726), Mogpoo, Silake, elevation 3000 m, 12 July 1884, s.c.|Boissier, P.E. (P00686490), Silake, elevation 609.6 m, *Buissier, P. E.* (P00686489). **Bangladesh.**24.25, 91.583, elevation 35 m, 1 March 1971, *Huq AM; Mia MK* (L0413463), elevation 40 m, 3 September 1999, 972 (E00189266). **Myanmar.** 13 August 1908, elevation 137 m, 4195 (E00097420). **Nepal.**26.816 N, 87.3 E, elevation 500 m, 13 August 1972, *1427* (E00500193). **United States of America. Maryland**: NMNH Botany Research Greenhouses. Suitland, 28 September 2001, *W. J. Kress & M. Bordelon 02-7123* (US3432988).

#### Taxonomic notes.

The new species is similar to two species in flower color, and absence of anther appendages. This species is different from *G.orixensis* in its petiolate leaves 0.5 mm (vs. subsessile), absence of inflorescence bracts (vs. present), short filament 1.8 cm (vs. 2.4 cm), and large anther 3.7 mm (vs. 2 mm). The species is different from *G.macroclada* in its petiolate leaves (vs. sessile), short inflorescence 3–8 cm (vs. 15 cm), and absence of inflorescence bract (vs. present). The detailed morphological comparisons between *G.tyrnaensis* and *G.orixensis* are presented in Table 3.

**Table 3.** Comparison of morphological characteristics of *G.tyrnaensis* sp. nov. and *G.janakiae* sp. nov. with their two closely related taxa. Characters not described in the original protologue or in subsequent descriptions of the same species are alternatively marked as not known.

Characters	*G.tyrnaensis* Y.Ritu & V.Gowda	*G.janakiae* Y.Ritu & V.Gowda	*G.orixensis* Roxb. (according to Darlong and Bhattacharyya 2011)	*G.macroclada* Gagnep.
Ligule length (mm)	3	1	1–3	2–3
Petiole (cm)	0.5	0.3	Subsessile	Sessile
Peduncle length (mm)	18	12	Not known	Not known
Inflorescence length (cm)	3–8	4–6	6.5	15
Rachis color and indumentum	Green, glabrous	Green, glabrous	Not known	Not known
Bract dimensions (mm) and color	Absent	Absent	Narrowly ovate, 5–7 × 2–3, acute at apex	Narrowly ovate, white or yellowish
Calyx length (mm)	8–9	8–9	5–10	8
Flower color	Orange	Orange	Orange yellow	Yellow
Labellum base	Decurrent on filament	Decurrent on filament	Not known	Not known
Lateral staminode dimensions (mm) and shape	4.7–4.8, narrowly ovate	3.2, ovate	Narrowly ovate	Narrowly ovate
Length of lateral staminodes with respect to corolla lobes	Shorter than corolla lobes	Shorter than corolla lobes	Shorter than corolla lobes	Equal
Filament length (cm)	1.8, orange	2, white- orange	2.4, yellow	Not known
Anther length (mm)	3.7	3.8	2	6–7
Anther crest	Yes	Yes	Not known	Not known
Ovary dimensions (mm)	3.1–3.6	3.4	Not known	Not known
Epigynous glands length (mm)	3	4	Not known	4
Fruit shape	Globose	Not observed	Globose	Not observed
Bulbil	Present	Present	Present	Absent
Bulbils shape	Ovoid	Narrowly ovate	Not Known	Linear

### ﻿ Globba janakiae

Taxon classificationPlantaeZingiberalesZingiberaceae

Y.Ritu & V.Gowda sp. nov.

A4562D47-86B9-5536-9DB9-42A487798948

urn:lsid:ipni.org:names:77347885-1

Fig. 10

#### Type.

**India. Meghalaya**: East Khasi Hills district, Tyrna village, Double decker bridge, 25.2513, 91.672, elevation 731 m, 25 July 2022, *Y. Ritu VG2022WB3727* (holotype: BHPL!; isotype: ASSAM!).

#### Diagnosis.

**Figure 10.**
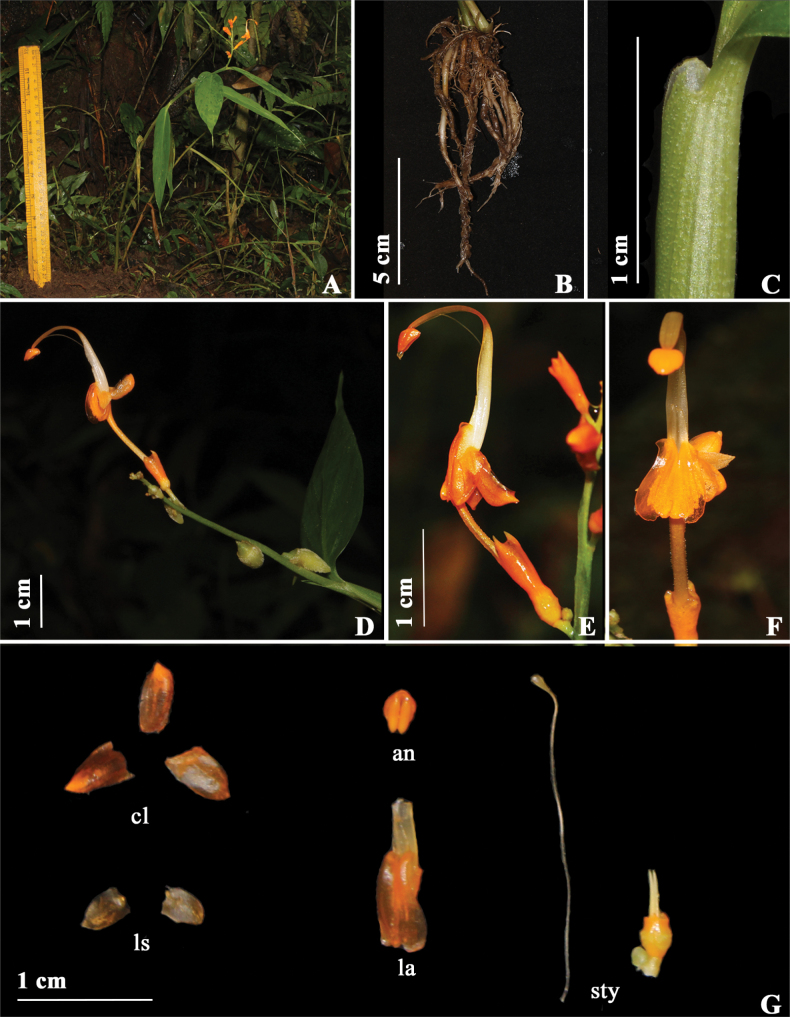
*Globbajanakiae* sp. nov. **A** habit **B** rhizome **C** ligule **D** inflorescence **E** flower (side view) **F** flower (front view) **G** dissected flower, cl (corolla lobes), an (anther), ls (lateral staminodes), la (labellum), sty (style with stigma and ovary with epigynous glands). Photographs by Y. Ritu.

*G.janakiae* is morphologically similar to *G.orixensis* and *G.macroclada* but different in having short inflorescence, absence of inflorescence bracts, petiolate lamina, short filament, large anther, heart-shaped labellum with cornicula (Fig. 10F) vs. large inflorescence, presence of inflorescence bracts, sessile lamina, large filament, small anther, and labellum without cornicula.

#### Description.

Terrestrial herbs, 40–46 cm tall including inflorescence height, pseudostem swollen at the base. Rhizomes compact, non-tuberulous, creamish-white. Leafy shoot with 8–10; sheath ligulate, ligule 1 mm long, bilobed, margin translucent and green in the center, pubescent; lamina 13 × 2.8 cm, subsessile, petiole 0.3 cm, green, glabrous, narrowly ovate, base obtuse, apex caudate, margin entire, glabrous. Inflorescence terminal to the leafy shoot, 4–6 cm long, erect; peduncle 12 mm long, green, glabrous; rachis, green, glabrous, bracts and bracteole absent. Ebracteate cincinni 6–8, glabrous, each cincinni with 1–2 flowers. Floral pedicel 0.1–0.5 cm long, light green, glabrous. Flowers 3–3.9 cm long, orange, recurved; calyx 8–9 mm long, yellowish orange; floral tube 0.5–0.6 cm long, dull orangish red, densely pubescent, curved upwards holding the flower upright; dorsal corolla lobe 6.6–7 × 2.5–3 mm, ovate, orange, densely pubescent; lateral corolla lobes 5.4 × 3 mm, ovate, apex acute, dark maroon with orange apex, glabrous; labellum 8.6 × 3 mm, cordate, corniculate, orange in center with light orange margins, labellum notch texture echinate, cornicula 0.3 mm in length, glabrous lateral staminodes 3.2 × 2.5 mm, ovate, orange, glabrous. Stamen 2.3 cm long, filament 2 cm long, orange, glabrous, arching; anther 3.8 mm long, ovate, apex truncate orange, crest 0.2–0.3 mm long, non-appendaged; style, filiform; stigma 4–5 mm, cylindrical, apex ciliate. Ovary 3.4 × 2.4 mm, obovate, pale green, verrucose; epigynous glands 2, 0.4 cm long, linear, cream. Fruits - not observed. Bulbils 1.2–2 × 0.5–0.8 cm, narrowly ovate, light green to light yellow, pubescent, bulbils present in inflorescence and axil of leaves.

#### Distribution and ecology.

At present, we have seen this species only in the Double decker bridge, Tyrna village, Cherrapunji, Meghalaya. We have observed 5–6 individuals in an overall area of ca. 400 m^2^. This species is terrestrial, mostly growing in understory habitats inside forests with an elevation of ca. 731 m. We have observed different species of bees visiting the flowers of this species.

#### Phenology.

Flowering was observed in the month of July.

#### Etymology.

The species is named in honor of Dr. E. K. Janaki Ammal. She was a pioneering Indian woman botanist who challenged the norms of caste, gender and race. She was critical about deforestation carried out to make way for development projects and advocated preservation of native plants.

#### Informal conservation status.

*Globbajanakiae* is currently known from one population in Double decker bridge (Cherrapunji district) within the state of Meghalaya, wherein the population was spread across 2–3 km along the edge of the road and has approximately 5–6 individuals. We surveyed seven potential locations in Meghalaya, which is 50 km^2^ around the type locality (Double decker bridge) and could not locate any population. Based on the IUCN (2022) guidelines, we hypothesize that if a formal conservation assessment were performed, its conservation status would probably be Critically Endangered according to criterias B1. (a) (B1: extent of occurrence is less than 100 km^2^, a: = 1 location) and D. (D: number of mature individuals <50).

#### Specimens of allied species examined.

***G.orixensis*: India. Assam**: Barak Valley, Cachar district, Kumbhirgram, 21 July 2009, Coll.: *D. Bhattacharyya 2501*, Fl. & Fr.; Dargakona, Assam University Silchar Campus, behind Life Science and Bioinformatics Department, along the trek path to ecoforest, 24.6870 N & 92.7521E, 13 July 2010, Coll.: Fl. (Herbarium of Department of Life Science & Bioinformatics, Assam University, Silchar), *L. Darlong & D. Bhattacharyya 10063*, 28 May 1808 (E00095817), 13 June 1920 (E00095807), (E00097427), elevation 610 m, 31 May 1882 (E00097430), 13 June 1920 (E00095807), elevation 610 m, 31 May 1882 (E00097430), 28 May 1808 (E00095817), 28 May 1809 (E00095821), (E00095820), (E00097427), (E00095812), 25.583 N, 91.633 E (L0487999), *Wallich N* (L0487998), Nayagarh, 5 August 1936, *H. F. Mooney 528* (L0487991). **Odisha**: Keonjhar, *H. F. Moooney 152* (L0488000). **Meghalaya**: Khasia, Regio Trop, *Hooker J. D.* (L0488002), Regio Trop, 28 September 2001, *Hooker J. D*. (P01743148) Hort Bot. Calcutta et Serampore, *Voigt, J.O. 101* (P00252266), East Himalaya, *5634* (P01743147). **Myanmar.** 13 August 1908 (E00097420), elevation 137 m, 13 August 1909 (E00097421), 13 August 1908 (E00097420), Sagaing Division, *W. J. Kress 2-7123* (US00605376). **Bangladesh.** Elevation 40 m, 3 September 1999 (E00189266), 22.695 N, 92.237 E, elevation 40 m, 5 September 1999 (E00189267), Sreemangal, Lawachera forest, 24.25 N, 91.583 E, 8 May 1997, *Williams K J* (L0413463)

***G.macroclada*: India.***Wallichn 6411* (L0487998), **Assam**: Khasia, 25.583 N, 91.633 E, 5 August 1936, *Tea Deputation Tea Deputation s. n.* (L0487999), Nayagarh, 8 May 1997, *Mooney H. F. 528* (L0487991). **Sikkim**: East Himalaya, *Griffith W 5639* (L0041113), *Herb. Watt 8702* (E00095812), (E00097427), *6536G* (E00095820), 28 May 1808, 7 (E00095821), elevation 610 m, 31 May 1882, *6931* (E00097430), 13 June 1920, 277 (E00095807), Khasia, *Hooker J. D.* (P01743148), East Himalaya, *Herb. Griffith 5634* (P01743147), Hort. Bot. Calcutta et Serampore, *Voigt, J. O. 101* (P00252266), East Himalaya, 12 July 1884, *Griffith, W. 5639* (P032726), Mogpoo, Silake, elevation 3000 m, 12 July 1884, s.c.|Boissier, P.E. (P00686490), Silake, elevation 609.6 m, *Buissier, P. E.* (P00686489). **Bangladesh.**24.25, 91.583, elevation 35 m, 1 March 1971, *Huq AM; Mia MK* (L0413463), elevation 40 m, 3 September 1999, 972 (E00189266). **Myanmar.** 13 August 1908, elevation 137 m, 4195 (E00097420). **Nepal.**26.816 N, 87.3 E, elevation 500 m, 13 August 1972, *1427* (E00500193). **United States of America. Maryland**: NMNH Botany Research Greenhouses. Suitland, 28 September 2001, *W. J. Kress & M. Bordelon 02-7123* (US3432988).

#### Taxonomic notes.

The new species is similar to two species in color of the flower, and absence of anther appendages. This species is different from *G.orixensis* in its petiolate leaves 0.3 mm (vs. subsessile), absence of inflorescence bracts (vs. present), and large anther 3.8 mm (vs. 2 mm). This species is different from *G.macroclada* in its short ligule 1 mm (vs. 2–3 mm), small size inflorescence 4–6 cm (vs. 15 cm), and short anther 3.8 mm (vs. 6–7 mm). Detailed morphological comparisons between *G.janakiae* and *G.orixensis* are presented in Table 3.

#### Notes.

**Figure 11.**
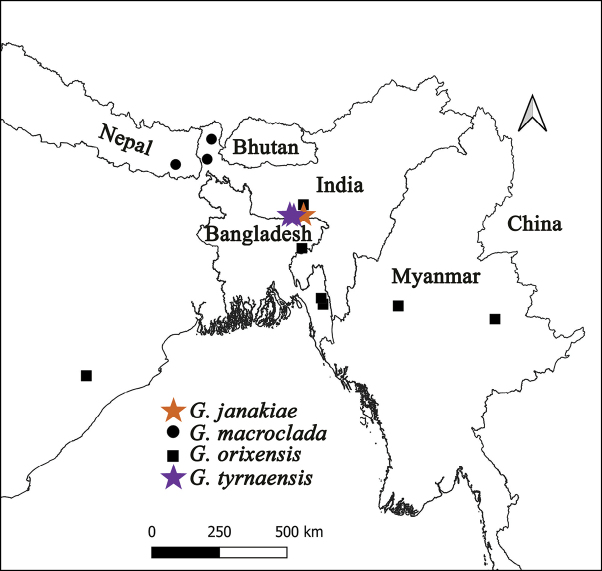
Map showing the distribution of *G.janakiae* sp. nov., *G.macroclada*, *G.orixensis*, *and G.tyrnaensis* sp. nov.

*G.macroclada* is distributed in Nepal and West Bengal, India, and *G.orixensis* is distributed in Bangladesh, Assam and Odisha states, India, and Myanmar (Fig. 11).

### ﻿ Globba yadaviana

Taxon classificationPlantaeZingiberalesZingiberaceae

Y.Ritu & V.Gowda sp. nov.

15AEF1ED-5AC1-5805-A48F-BAB41291A8CD

urn:lsid:ipni.org:names:77347887-1

Fig. 12

#### Type.

**India. Mizoram**: Mamit district, Reiek Tlang road, 23.6777, 92.6037, elevation 300 m, 28 September 2022, *Y. Ritu & P. A. Shangreiphao VG2022MZ3958* (holotype: BHPL!; isotype: ASSAM!).

#### Diagnosis.

**Figure 12.**
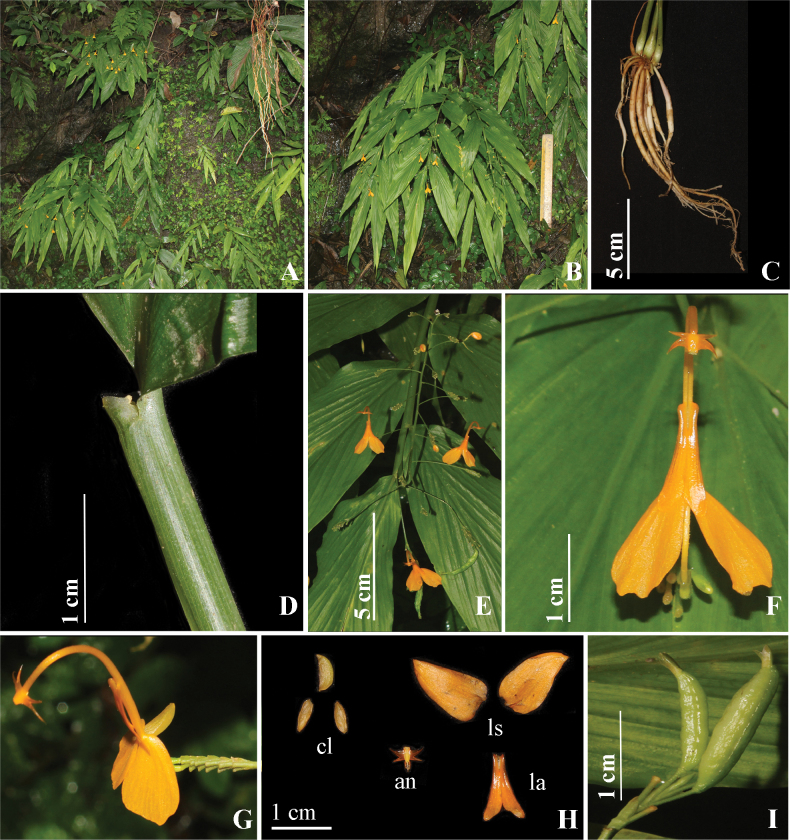
*Globbayadaviana* sp. nov. **A** habitat **B** habit **C** rhizome **D** ligule **E** inflorescence **F** flower (front view) **G** flower (side view) **H** dissected flower, cl (corolla lobes), an (anther), ls (lateral staminodes), la (labellum) **I** fruit. Photographs by Y. Ritu.

*Globbayadaviana* is morphologically similar to *G.rahmanii*, *G.expansa* and *G.lancangensis* but clearly different in pod shape fruit (Fig. 12I), absence of andromonoecy, reduced peduncle, large inflorescence, short calyx vs. oblong, globose ellipsoid fruit, presence of andromonoecy, large peduncle, small inflorescence, and large calyx.

#### Description.

Lithophytic herbs, 35–70 cm tall including inflorescence height, pseudostem swollen at the base. Rhizomes compact, non-tuberulous, creamish-white. Leafy shoot with 5–10 leaves; sheath ligulate, ligule 4–5 mm long, bilobed, light green, sparsely pubescent; lamina 26–35 × 6.5–11 cm, sessile, narrowly ovate, base obtuse, apex caudate, margin entire, glabrous. Inflorescence terminal to the leafy shoot, 6–35 cm long, erect; peduncle reduced, light green, glabrous; rachis, green, pubescent; bracts 2.3–2.5 × 0.7–0.8 mm, elliptic, green, pubescent and bracteoles 2.5–2.6 × 1–1.3 mm, ovate, light green, glabrous. Cincinni 5–39, each cincinni with 7–18 flowers. Floral pedicel for basal flowers 2.5–4 cm long, for terminal flowers 0.6 –1.5 cm long, light green, pubescent. Flowers 3–3.6 cm long, yellow-orange, recurved; calyx 2–2.5 mm long, grayish green; floral tube 3.2 mm long, yellow, densely pubescent, slightly curved at the middle; dorsal corolla lobe 6.8 × 2.6–2.8 mm, ovate, yellow tinged green, glabrous; lateral corolla lobes 4.8–4.9 × 2.2 mm, ovate, apex obtuse, yellow green tinged, glabrous; labellum 11.5 × 3 mm, sagittate, yellow, labellum notch texture echinate; lateral staminodes 14–15 × 5.5–5.6 mm, ovate, apex acute, yellow, hairs on margins and on veins. Stamen 2.2 cm long; filament 2 cm long, yellow, glabrous, arching; anther 1.9–2 mm long, elliptic, apex truncate, yellow, crest absent, appendaged; style, filiform, tip broader than base, stigma 4–5 mm wide, clavate, ciliate. Ovary 0.4–0.5 × 0.2–0.3 cm, obovate, pale green, verrucose; epigynous glands 2, 0.4–0.5 cm long, linear, cream. Fruit 1.5 cm in length, linear, base obtuse, green, texture undulate, calyx persistent. Bulbils absent.

#### Distribution and ecology.

At present, we have seen this species in Reiek Tlang road, Mamit, Mizoram. We have observed ca. 30 individuals in an overall area of ca. 400 m^2^. This species is lithophytic, mostly growing along the edge of roads with an elevation of ca. 300 m. We have observed different species of bees and butterflies visiting the flowers of this species. We have observed ants as seed dispersal for this species.

#### Phenology.

Flowering and fruiting were observed in the month of August.

#### Etymology.

This species epithet is in the memory of the late Mr. Rajesh Yadav, who was the father of the first author and was instrumental in the author’s progress in science and education.

#### Informal conservation status.

This species is currently known from 4–5 small populations across Reiek-Tlang road (Mamit district) within the state of Mizoram, wherein each population is spread across 1–2 km along the edge of the road and has approximately 30 individuals each. Although this species is known only from the type locality, it could be more widespread in neighboring countries with similar habitats, we, therefore, assess it as Data Deficient (DD).

#### Specimens of allied species examined.

***G.rahmanii*: Bangladesh. Khagrachari**: Dighinala- Marissha road, Teentila, 30 August 1997, *Rahman et al., 1878* (HCU)

***G.expansa*: India. Assam**: *Jenkins F, Jenkins, F s.n.* (L0487987), 1 May 1908, *Alleizette AC d’, Alleizette, AC d’ 7115* (L0488012), 1 July 1909, *Alleizette AC d’. Alleizette AC d’ s.n.* (L0487993), 12 June 1910 (E00097428), *Tea Deputation* (NYBG04355596). **West Bengal**: Hortus Botanicus Calcuttensis, 22.559 N, 88.291 E, *Anon 6536E* (BM013718590), Circa Calcuttam, *J. W. Helfer 192* (NYBG04355597). **Thailand. Chiang Mai**: Mae Rim, 19.166 N, 98.833 E, elevation 700 m, 8 August 1990, *J. F. Maxwell 89-912* (L0413531), Mua ng, 18.833 N, 98.883 E, elevation 400 m, 3 July 1992, *J. F. Maxwell 90-847* (L0413571), Maerim, 19.166 N, 98.833 E, elevation 700 m, 15 September 1995, *J. F. Maxwell 92-358* (L0413518), Doi Intanon, 18.533 N, 98.566 E, elevation 1100-1200 m, 17 May 1995, *Larsen K; Larsen SS; Tange C; Sookchaloem D, 46485* (L0413547), *Sahngahmpang, Mae Awn*, elevation 1050 m, 31 May 2006, *J. F. Maxwell 95-403* (L0413491), Doi Suthep-Pui National Park, elevation 960 m, *A.F.G. Kerr* (E00097516), 18.55 N, 98.6 E, elevation 710 m, 19 September 2008, *4513* (E00533779), 18.855 N, 100.734 E, elevation 300 m, 16 August 2012, *5614* (E00680827), *Fleuve Petchaponai, Pierre, L.* (P00234360), *Neeckey près Wangka*, elevation 150 m, 9 May 1946, *Hoed, G. den 246* (P00234428), elevation 10 km *W Fang*, 19.95 N, 99.183 E, elevation 600 m, 24 July 968, *Larsen, K. Santisuk, T.Warncke, E. 2650* (P00234440), Bo Luang, 18.75 N, 98.416 E, elevation 1050 m, 12 June 1973, *Geesink, R. Phanichapol, D. Santisuk, T. 5886* (P00234419), Mai Rim, Nae Rin, Doi Sutop Pui Natl. Park, elevation 700 m, 3 July 1992, *J. F. Maxwell 92-356* (P00234313), Doi Suthep, elevation 975.36 m, 12 June 1910, *1214* (BM013718611), Ang-ka-noi, 26 June 1978, *C. Phengklai, 4121* (NYBG04347253). **Lao People’s Democratic Republic. Khammouan**: Laos P.D.R., 18.394 N, 103.075 E, elevation 199 m, *Newman MF; Thomas PI; Armstrong KE; Lamxay V; Sengdala K LAO-1529* (L0811897), Laos, 14.949 N, 106.886 E, elevation 105 m, 15 July 2009, *VL1957* (E00640106), Phabat, 18.3944 N, 103.0758 E, elevation 199 m, 31 May 2006, *Newman, M.F., Thomas, P., Armstrong, K., Lamxay, V., Sengdala, K. LAO1529* (P01743264), Luang Prabang, Mekong river, 19.8669 N, 102.0630 E, elevation 303 m, 12 June 2012, *Jana Leong-Skornickova; Tran Huu Dang; Ota Sida; Kittisack Phoutthavong; Somdy Oudomsack JLS1688* (P00840173). **Cambodia. Mondulkiri**: 13.429 N, 103.763 E, elevation 491 m, 2 November 2006, *Long, C. Cheng, K.C. Leti, M. CL435* (P00626289)

***G.lancangensis*: China.** 22.061 N, 100.194 E, elevation 1210 m, 14 July 2000, *00-253* (E00187763), 22.543 N, 99.943 E, elevation 1080 m, 13 July 2000, *00-241* (E00187762).

#### Taxonomic notes.

The new species is similar to three species in flower color, and presence of four anther appendages. This species is different from *G.rahmanii* in its large inflorescence 6–35 cm (vs. 8–19 cm), small calyx 2–2.5 mm (vs. 3.5–5 mm), large ovary 3.5–3.7 mm (vs. 1 mm) and dorsal corolla lobes with a keel (vs. without a keel). This species is different from *G.lancangensis* and *G.expansa* in its inflorescence length 6–35 cm (vs. 10–27 cm), small calyx 2–2.5 mm (vs. 4 mm), absence of anther crest (vs. present), and pod shape fruit (vs. globose and ellipsoid). The detailed morphological comparisons between *G.yadaviana* and *G.rahmanii*, *G.lancangensis*, *G.expansa* are presented in Table 4.

**Table 4.** Comparison of morphological characteristics of *G.yadaviana* sp. nov. with *G.rahmanii*, *G.lancangensis* and *G.expansa*. Characters not described in the original protologue or in subsequent descriptions of the same species, are marked as not known.

Characters	*G.yadaviana* Y.Ritu & V.Gowda	*G.rahmanii* Yusuf (Yusuf 2004)	*G.lancangensis* Y.Y.Qian (according to Sangvirotjanapat et al. 2019)	*G.expansa* Wall. (according to Sangvirotjanapat et al. 2019)
Ligule length (mm)	4–5	3	2	1–6
Peduncle length (cm)	Absent	Not known	1.5–3	3
Inflorescence length (cm)	6–35	8–19	10–20	10–27
Rachis color and indumentum	Green, densely pubescent	Green, pubescent	Light green, minutely pubescent;	Green, glabrous
Bract dimensions (mm) and color	2.3–2.5 × 0.7–0.8, elliptic, green, pubescent	2–5–3 × 2, ovate, green, sparsely hairy	1.5 × 1–2, caducous	10, caducous
Calyx length (mm)	2–2.5	3.5–5	4	4
Flower color	Yellowish orange	Yellow	Primrose or pale yellow	Yellowish orange
Lateral staminode dimensions (mm)	14–15 × 5.5–5.6	13–19 × 9–10	10–13 × 4–5	11–12 × 4–5
Dorsal corolla lobe keel	Present	Absent	Present	Present
Filament length (mm)	20	18	23	26–28
Anther length (mm)	1.9–2	2	2	1–2
Crest length (mm)	Absent	Not known	1	1
Ovary dimensions (mm)	3.5–3.7	1	3	4
Fruit shape	Pod shape, more linear with undulate margins	Oblong, with smooth margins	Globose	Ellipsoid
Bulbil	Absent	Absent	Absent	Bulbils occasionally

#### Notes.

**Figure 13.**
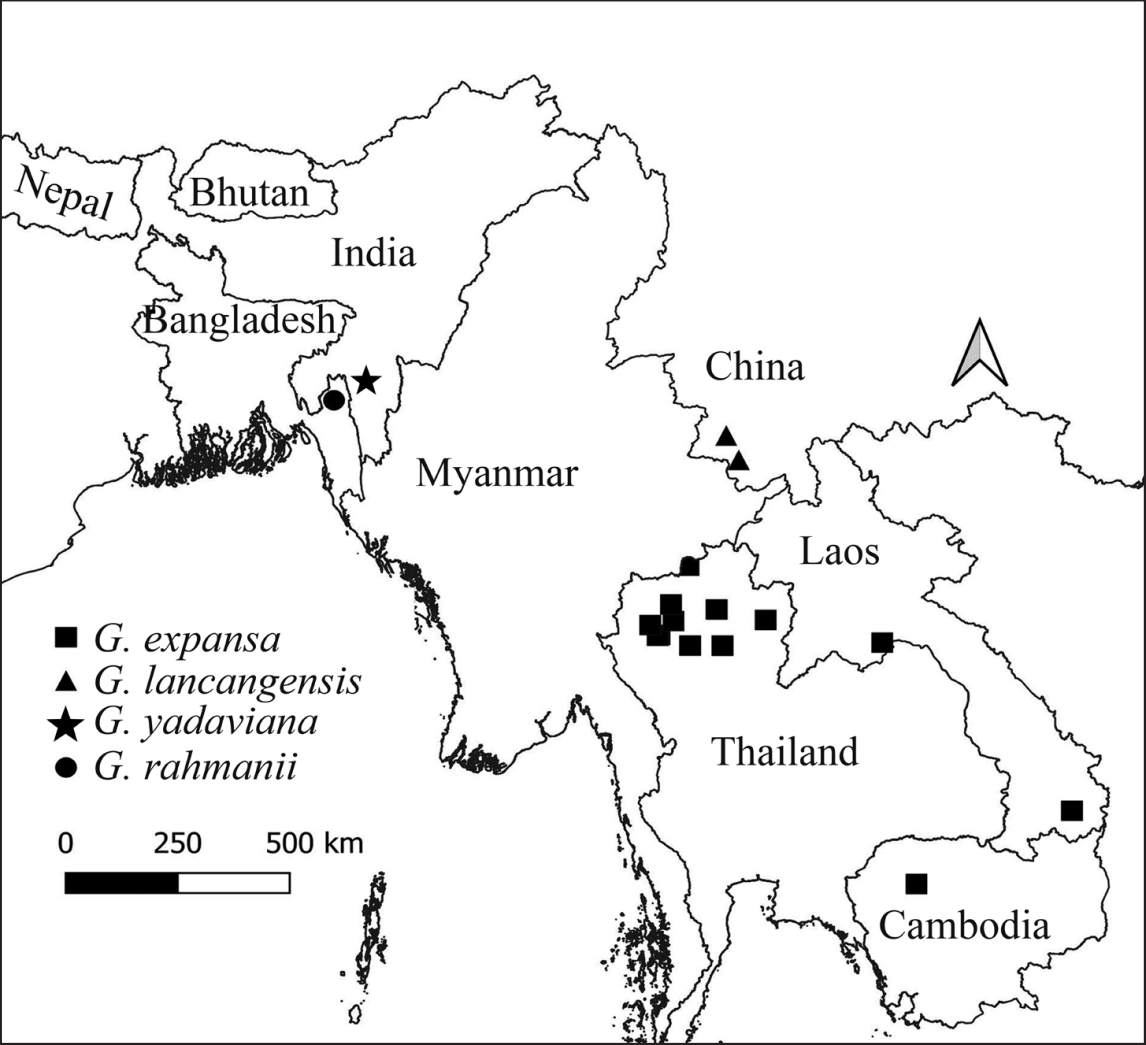
Map showing the distribution of *G.expansa*, *G.lancangensis*, *G.yadaviana* sp. nov., and *G.rahmanii*.

*G.rahmanii* is distributed to Bangladesh, *G.lancangensis* is distributed to China, and *G.expansa* is distributed to Thailand, Laos, and Cambodia (Fig. 13).

##### ﻿Taxonomic key to the Indian *Globba* species

**Table:** 

1	Prominent bracts; anther appendages present	**2**
–	Not prominent bracts; anther appendages absent	**12**
2	Bracts large imbricating then spreading; 4 anther appendages	**3**
–	Bracts small not imbricating; 2 anther appendages	**7**
3	Pendant inflorescence; red spot at the center of labellum	**4**
–	Erect inflorescence; red spot absent at the center of labellum	**5**
4	Leaves elliptic or ovate/narrowly ovate; inflorescence bracts oblong; green	***G.schomburgkii***
–	Leaves elliptic; inflorescence bract narrowly ovate; white	***G.sherwoodiana***
5	Bracts persistent; ovate; bulbils present	***G.marantina***
–	Bracts deciduous; bulbils absent	**6**
6	Leaves oblong; inflorescence short (<5 cm); cincinni lax with few flowers	***G.pauciflora***
–	Leaves narrowly ovate; inflorescence long (6–35 cm); cincinni crowded with numerous flowers	***G.yadaviana***
7	Inflorescence pendent; lateral staminodes attached below labellum	**8**
–	Inflorescence erect; lateral staminodes attached above labellum	**9**
8	Leaves oblong; bulbils absent	***G.bracteolata***
–	Leaves narrowly ovate; bulbils present	***G.pendula***
9	Flowering precedes the onset of vegetative shoot	**10**
–	Flowering occurs on vegetative shoot	**11**
10	Bracteole light pink; flowers generally white; labellum yellow	***G.spathulata***
–	Bracteole light green; flowers yellow; labellum yellow	***G.wengeri***
11	Leaves narrowly ovate; bracts ovate; purple; floral tube yellow; lateral staminodes facing upwards	***G.radicalis***
–	Leaves cardio-acuminate; bracts elliptic; violet; floral tube reddish purple; lateral staminodes facing downwards	***G.wardii***
12	Ovary long (>0.3 cm); bulbils present	**13**
–	Ovary small (<0.3 cm); bulbils absent	**22**
13	Inflorescence bracts persistent; flower bracteole present	**14**
–	Inflorescence bracts caducous or absent; flower bracteole absent	**15**
14	Flower yellow; lateral staminodes erect; backwardly positioned	***G.kanchigandhii***
–	Flower white; lateral staminodes not erect; downward positioned	***G.andersonii***
15	Inflorescence short (<12 cm); latera staminodes short (<0.5 cm) cornicula present	**16**
–	Inflorescence long (>12 cm); lateral staminodes long (>0.5 cm); cornicula absent	**18**
16	Labellum obcuneate; labellum saffron yellow with 2 red blotches	***G.multiflora***
–	Labellum not obcuneate; labellum without red blotch	**17**
17	Ligule 3 mm; labellum ovate; lateral staminodes narrowly ovate	***G.tyrnaensis***
–	Ligule 1 mm; labellum cordate; lateral staminodes ovate	***G.janakiae***
18	Fruit smooth; bulbils only in the axil of leaves; lateral staminodes longer than corolla lobes	***G.macroclada***
–	Fruit warted; bulbils produced on the inflorescence; lateral staminodes equal to corolla lobes	**19**
19	Calyx brown; floral tube yellow with a tinge of brown; bulbils produced on the upper part of inflorescence	***G.clarkei***
–	Calyx not brown; floral tube yellow; bulbils produced all over the inflorescence	**20**
20	Inflorescence crowded; leaves oblong; labellum narrowly obovate with outwards curled; lateral staminodes longer than corolla lobes	***G.sessiliflora***
–	Inflorescence lax leaves not oblong; labellum not narrowly obovate with outwards curled; lateral staminodes equal to corolla lobes	**21**
21	Inflorescence long (>10 cm); calyx dark purple; flower yellow	***G.racemosa***
–	Inflorescence up to 6.5 cm; calyx yellow, flower orange yellow	***G.orixensis***
22	Ligule with unequal lobes; labellum sagittate; off-white with orange spot at center	***G.paschimbengalensis***
–	Ligule with equal lobes; labellum linear; orange with no orange spot in center	***G.polymorpha***

One species, *G.platystachya*, is not included in the above taxonomic key since anther characters were not described in its protologue, and anther characters are very critical for subgeneric classification in *Globba*. To the best of our knowledge there has been no subsequent record of this species. Since the description of this species is incomplete, we did not include it in this dichotomous key.

##### ﻿A note on the taxonomic collection challenges in the ecologically sensitive Eastern Himalayas and northeast region of India, and recommendations:

In the past few decades, the Eastern Himalayas and the Northeast India are two biodiversity-rich regions that have been constantly threatened by rapid development projects. The effects of ecological destruction caused in this region need special mention and attention because these regions are among the least explored areas in India for their flora, fauna, ecology, and evolutionary patterns, and they remain one of the most challenging regions for collection-based studies, both logistically and politically. Based on our decade-long experience of working in this region, we discuss below two main challenges in collection-based floristic and taxonomic studies: 1) access to study areas and associated logistic challenges, and 2) safety in the field.

**Figure 14.**
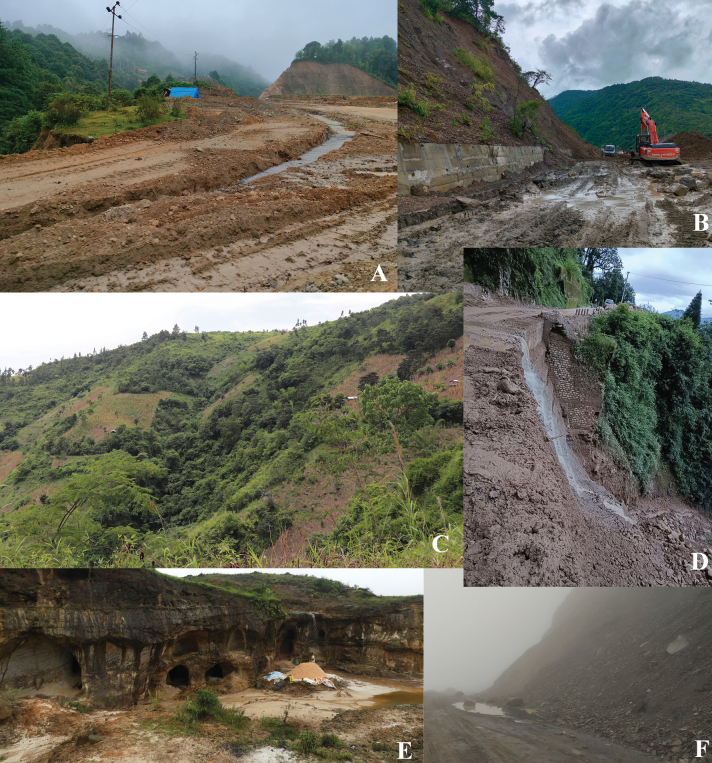
Images depicting common logistic hurdles in field collections when working in the Northeast states of India **A** Willong Road, Manipur **B** mining in Cherrapunji road, Meghalaya **C** forest clearing, on the way to Thanamir, Nagaland **D** stone cutting Ziro road, Arunachal Pradesh **E** Mawphlang Sacred Grove, Meghalaya **F** roadblock because of a landslide in Lachen, Northern Sikkim. Photographs **A, D** by Ajith Ashokan **B** by N S Prasanna **C** by Aleena Xavier **E, F** by Y. Ritu.

**1) *Access to study areas***—The only mode of transportation within Northeast India is by road, with a single rail line that connects Guwahati (Meghalaya) to Tinsukia (Arunachal Pradesh). The roadways within Northeast states are well-connected, but they are difficult to traverse due to poor road quality, and unpredictable landslides that can completely isolate large parts of this region for an indefinite time (Fig. 14). Lastly, the lack of frequent public transportation means that fieldwork can only be carried out using privately hired vehicles, which significantly increases fieldwork budgets.

One of the forest types that is challenging to access in the northeastern states is the sacred groves (Fig. 14E). Sacred groves cover over 40000 hectares of natural forest area across five states (Upadhyay et al. 2019) and they represent a valuable practice in conserving biodiversity, where local communities preserve a specific site untouched out of reverence for religious or cultural reasons. This conservation method is essential in protecting threatened flora and fauna from extinction as well as preserving socio-ecologically important species. While it is possible to access most sacred groves via research permits from the indigenous guardian communities, collecting samples within the groves is restricted. This presents a significant challenge in identifying species from these sacred groves since voucher collections from these locations are prohibited.

Other logistic challenges include scarcity of paid accommodations and access to basic amenities such as electricity for drying herbarium specimens. The high humidity in this region makes it difficult to dry specimens, especially in areas like Cherrapunji, Meghalaya, where rainfall can reach up to 450 inches (11430 mm). Finally, the absence of a continuous electrical power source also means that charging of field equipment such as cameras, GPS devices, power banks, and mobile phones becomes challenging here.

**2) *Safety in the field***—The safety of researchers has been a major concern in the northeastern states due to the combination of natural disasters and political conflicts. This region shares political boundaries with five countries, making it a politically sensitive area, especially in regions closer to the political borders.

The Northeast states are biodiversity corridors between the Indian subcontinent and Southeast Asia (Ashokan et al. 2022, Prasanna et al. 2020). With the region’s needs to focus on development, it is very critical to take active measures in documenting the biodiversity present in this area. Our discovery of six new species highlights the importance of documenting and preserving the flora of this biodiverse region. Here, we have briefly discussed the research challenges in this region to highlight and emphasize the need for an active dialogue towards building local facilities for taxonomic studies such as botanical gardens, herbaria, and tissue collections. Based on our studies in the northeast region we suggest that a collective effort from local communities, researchers, taxonomists, forestry personnel, and policymakers are the need of the hour in order to mitigate biodiversity loss in this region.

## Supplementary Material

XML Treatment for Globba corniculata

XML Treatment for Globba paschimbengalensis

XML Treatment for Globba polymorpha

XML Treatment for Globba tyrnaensis

XML Treatment for Globba janakiae

XML Treatment for Globba yadaviana

## ﻿Additional information

### Author ORCIDs

Ritu Yadav https://orcid.org/0009-0001-3317-183X

Vinita Gowda https://orcid.org/0000-0001-8533-0014

### Data availability

All of the data that support the findings of this study are available in the main text or Supplementary Information.

## Supplementary materials

Supplementary material 1 Type localities of Indian *Globba* spp. Data type csv Explanation note Table contains the occurence data for type localities of all 19 Indian *Globba* spp. https://binary.pensoft.net/file/1128072This dataset is made available under the Open Database License (http://opendatacommons.org/licenses/odbl/1.0/). The Open Database License (ODbL) is a license agreement intended to allow users to freely share, modify, and use this Dataset while maintaining this same freedom for others, provided that the original source and author(s) are credited.Ritu Yadav, Vinita Gowda
